# Fasting During Chemotherapy and Hematologic, Metabolic, and Quality of Life Outcomes: A Systematic Review and Meta-Analysis

**DOI:** 10.3390/nu18213681

**Published:** 2026-11-06

**Authors:** LaShae D. Rolle, Harleen Kaur, Cameron G. Jacobsen, Melanie E. Stone, Dominique Thomas, Kate M. Bonilla, Loren Yavelberg, Jaime Rafael Merchan, Soyeon Ahn, Tracy E. Crane

**Affiliations:** 1 Division of Prevention Science and Community Health, Department of Public Health Sciences, Miller School of Medicine, University of Miami, Miami, FL 33136, USA; 2 Miller School of Medicine, University of Miami, Miami, FL 33136, USAcgj37@med.miami.edu (C.G.J.);; 3 Sylvester Comprehensive Cancer Center, Division of Medical Oncology, University of Miami, Miami, FL 33136, USA; 4 VCU Massey Comprehensive Cancer Center, Virginia Commonwealth University, Richmond, VA 23298, USA; 5 Department of Psychiatry, School of Medicine, Virginia Commonwealth University, Richmond, VA 23298, USA; 6 Department of Psychology and Medicine, Division of Medical Oncology, Miller School of Medicine, University of Miami, Miami, FL 33136, USA; 7 School of Education and Human Development, University of Miami, Miami, FL 33136, USA

**Keywords:** fasting, chemotherapy, quality of life, side effects, review, meta-analysis

## Abstract

Background/Objectives: Fasting has been proposed as a non-pharmacologic method of reducing chemotherapy toxicity. However, its effects across outcome domains have not been well-quantified. Methods: In line with PRISMA 2020 guidelines (PROSPERO: CRD420261296778), we searched PubMed, Embase, Web of Science, and CENTRAL from January 2015 through April 2026 for randomized or prospective comparative trials evaluating fasting during chemotherapy. Risk of bias was assessed using RoB 2 and criteria aligned with ROBINS-I. Standardized mean differences (Hedges’ g) and risk ratios (RRs) were pooled using three-level random-effects models. Results: Seven publications reporting six trials (268 participants) were included. The pooled estimate did not reach statistical significance (g = 0.21, 95% CI: −0.02–0.43, *p* = 0.064). Hematologic parameters (g = 0.11, 95% CI: −0.23–0.45) and quality of life (QoL) and patient-reported outcomes from one trial (g = 0.32, 95% CI: −0.03–0.66) did not differ significantly between groups. Adverse events also did not differ significantly (RR = 0.86, 95% CI: 0.55–1.36). Conclusions: Across six small trials, fasting during chemotherapy did not significantly affect hematologic parameters, QoL, or adverse events. However, it is worth nothing that point estimates generally favored fasting. Hence, a small benefit can neither be confirmed nor ruled out at this time. Adequately powered trials assessing patient-important outcomes and incorporating nutritional monitoring are needed before fasting can be recommended or ruled out.

## 1. Introduction

Cancer continues to be a leading global health burden, with 20.0 million new cases and 9.7 million deaths reported worldwide in 2022 [1]. Despite therapeutic advances, chemotherapy-induced toxicity affects approximately 45% of the cancer population receiving treatment, with grade 3–5 events including substantial hematologic impairment (anemia in 90% and febrile neutropenia in 7% of cases) [2]. These toxicities compromise treatment completion, diminish quality of life (QoL), and contribute to the $208.9 billion annual US cancer care expenditure [3]. Given the persistent burden of treatment-related toxicity and its downstream effects on treatment delivery, quality of life, and healthcare costs, there is growing interest in low-cost supportive care strategies that may improve treatment tolerability without compromising therapeutic efficacy. Short-term fasting (STF) is defined as brief periods of complete or near-complete caloric restriction, typically lasting 24–72 h surrounding chemotherapy administration [3,4,5]. Fasting-mimicking diets (FMDs) are low-calorie (approximately 300–1100 kcal/day), low-protein, low-sugar dietary regimens designed to replicate the metabolic effects of fasting while allowing limited food intake, typically administered for 3–5 days during chemotherapy cycles [6,7,8,9]. Intermittent fasting (IF) encompasses various cyclical patterns of fasting and eating, including time-restricted eating (TRE), which limits food intake to defined windows (e.g., 6–12 h per day), alternate-day fasting, or the 5:2 diet involving fasting 2 days per week [10,11]. Water-only fasting (WOF) entails complete caloric restriction with only water consumption, typically for 24–48 h before and/or after chemotherapy [12,13]. Short-term caloric restriction (SCR) similarly involves water-only fasting periods of approximately 48 h repeated across multiple treatment cycles [14]. All of these approaches are grounded in the differential stress resistance (DSR) hypothesis, which argues that fasting-induced metabolic stress selectively protects normal cells while increasing the susceptibility of cancer cells to chemotherapy-related toxicity [15,16].

Raffaghello et al. (2008) demonstrated that 48–72 h fasting triggers divergent responses wherein normal cells enter a self-protective state with reduced proliferation, while cancer cells (driven by constitutive oncogenic signaling) fail to downregulate growth pathways, becoming paradoxically more vulnerable to chemotherapy [15]. This phenomenon is mediated through reduced insulin-like growth factor-1 (IGF-1) signaling, with fasting decreasing IGF-1 by 70% with a 72 h fast while increasing IGF-binding protein-1 11-fold [17]. Reduced IGF-1/PKA signaling promotes hematopoietic stem cell regeneration and reverses chemotherapy-induced immunosuppression [18]. Preclinically, 48 h STF combined with chemotherapy achieved complete tumor regression in 20–40% of mice with aggressive neuroblastoma, whereas neither intervention alone (chemotherapy or fasting) was curative [14].

Early clinical studies were small and largely uncontrolled. A 2009 case series of 10 patients who voluntarily fasted for 48 to 140 h around chemotherapy reported fewer self-reported gastrointestinal side effects [5], and a dose-escalation study of fasting for up to 72 h around platinum-based chemotherapy reported less nausea and vomiting with longer fasting [4]. In a pilot randomized controlled trial (RCT), de Groot et al. (2015) reported preserved erythrocyte and thrombocyte counts and less chemotherapy-induced DNA damage in immune cells with short-term fasting [19], and in the multicenter DIRECT trial (n = 131), a FMD was associated with higher radiological and pathological response rates in HER2-negative breast cancer [7]. A crossover study reported better-preserved QoL and less fatigue with 60 h fasting [20]. Narrative reviews have summarized this evidence [10,12], but the underlying studies were small, used different fasting protocols, and measured different outcomes.

Despite accumulating evidence, no systematic synthesis has quantified the effects of fasting across hematologic, metabolic, and QoL domains. This meta-analysis aims to evaluate fasting effects on these outcome domains in cancer patients receiving chemotherapy.

## 2. Methods

### 2.1. Design and Registration

This meta-analysis examined the effects of fasting-based interventions including FMDs, STF, WOF, IF, and SCR on clinical outcomes and patient-reported QoL among adults receiving chemotherapy. The review followed Preferred Reporting Items for Systematic Reviews and Meta-Analyses (PRISMA) 2020 guidance and was prospectively registered on PROSPERO (CRD420261296778). The protocol prespecified the research question, eligibility criteria, outcomes of interest, synthesis methods, and planned sensitivity analyses.

### 2.2. Eligibility Criteria

Eligible studies were RCTs, quasi-randomized trials, cluster-randomized trials, prospective comparative cohort studies, or non-randomized matched-pair studies with identified comparators, that enrolled adults aged 18 years or older with histologically or clinically confirmed malignancy undergoing active systemic treatment. Studies were required to implement a fasting regimen (i.e., FMD, STF, WOF, IF, or SCR, including modified protocols permitting limited rescue caloric intake) and include a comparator group of non-fasting counterparts concurrent with treatment delivery, as well as report at least one validated outcome measure, including hematologic parameters, metabolic biomarkers, treatment-related toxicity, tumor response, or patient-reported assessments using validated instruments.

Secondary analyses of included trials were eligible when reporting non-overlapping outcomes and were explicitly linked to the parent trial to prevent double counting of participants or effect sizes. In the synthesis, a secondary analysis and its parent trial were treated as a single cohort (Section 2.7). Eligible studies were published in English and included all geographic regions, provided that full-text articles were available in English, and reported extractable summary statistics, including means and standard deviations for continuous outcomes or event counts for dichotomous outcomes.

Studies were excluded if they were single-arm observational studies, retrospective cohorts without matched comparators, focused exclusively on cancer survivors in the post-treatment stage rather than patients undergoing active treatment, were preclinical or animal studies, were conference abstracts without sufficient data, or lacked validated outcome measures.

### 2.3. Information Sources and Search Strategy

A comprehensive search was initially conducted for records published from 1 January 2015, through 30 September 2025, across four electronic databases: PubMed, Embase via Ovid, Web of Science Core Collection, and CENTRAL. An updated search was subsequently conducted on 23 April 2026, across the same four databases, to capture the literature published after the initial search period. The search combined, with the Boolean operator AND, terms for cancer; fasting exposures (fasting, time-restricted eating or feeding, FMDs, intermittent fasting, alternate-day fasting, short-term fasting, and intermittent energy restriction); intervention and comparative study designs; and outcomes (treatment outcomes, quality of life, fatigue, progression-free survival, CTCAE, adverse events, and treatment completion rates). Records on post-treatment or survivorship-only populations and preclinical or animal studies were excluded with the operator NOT. We used free-text terms in PubMed, Web of Science, and CENTRAL, and added Emtree terms with explosion in Embase. The full search strings for each database are reported in Supplementary Table S2.

### 2.4. Study Data Collection

Retrieved records were imported into Covidence systematic review software (https://www.covidence.org, accessed on 29 August 2025) and deduplicated. Title and abstract screening employed SWIFT-Review for machine learning-assisted prioritization with manual verification [21]. Two reviewers independently screened titles and abstracts for each manuscript, with contributions from the project team (K.M.B., C.G.J., M.S., D.T., H.K., L.Y., L.D.R.), with disagreements resolved through group discussion. Full-text screening was conducted independently by two reviewers from the project team (K.M.B., C.G.J., M.S., D.T., H.K., L.Y., L.D.R.) using standardized eligibility forms, with disagreements resolved through group discussion or consultation with a third reviewer (T.E.C.). Data extraction was performed independently by two reviewers for each manuscript using structured forms to capture study identifiers, design characteristics, sample characteristics, intervention details, comparator descriptions, outcome instruments and assessment timing, risk of bias elements, and numeric data for meta-analysis. Nutritional eligibility criteria, changes in body weight or body mass index (BMI), adherence, and fasting-related adverse effects were also extracted from each trial report (Supplementary Table S4).

### 2.5. Study Selection and Characteristics

**Figure 1:**
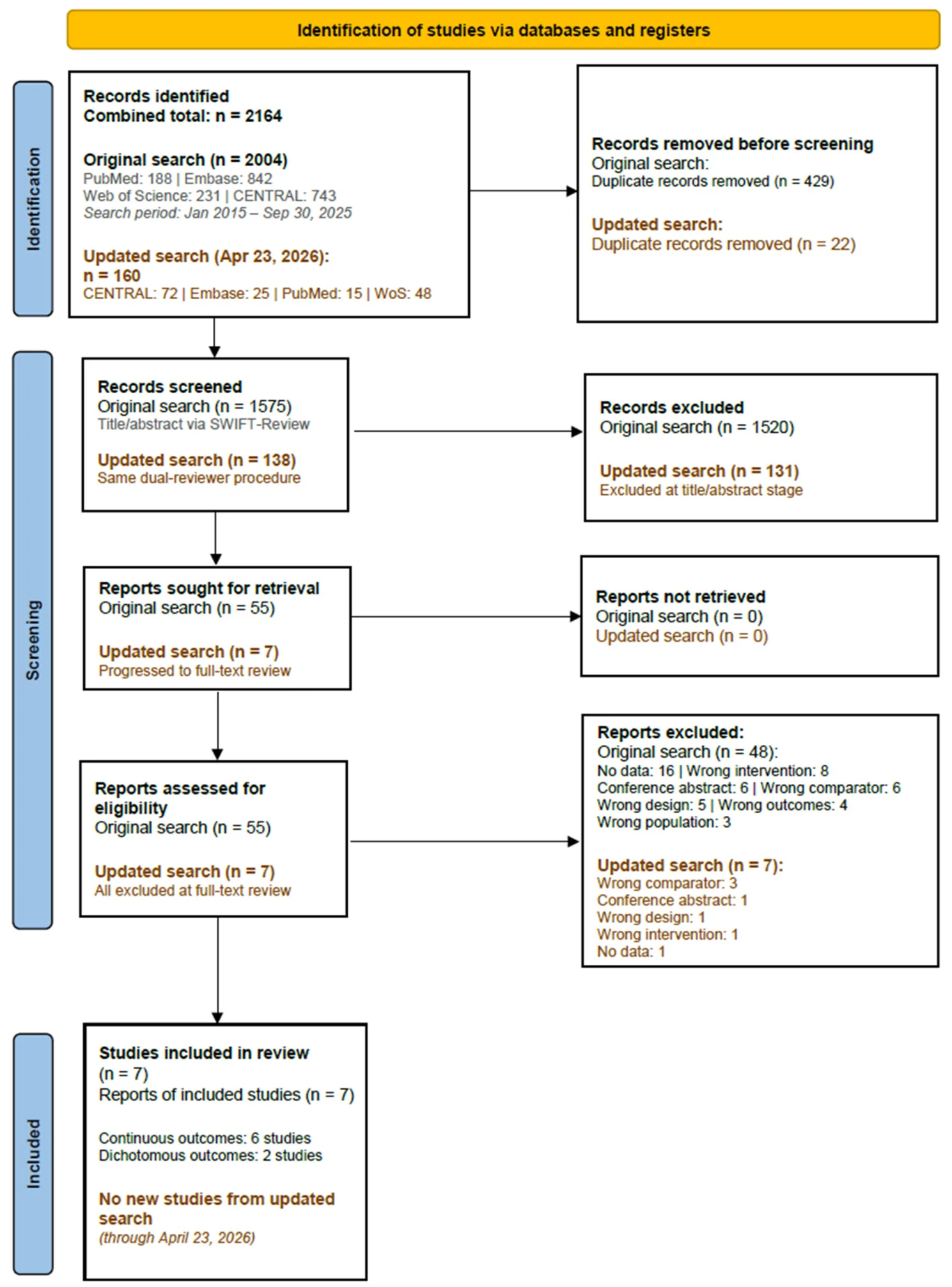
PRISMA 2020 Flow Diagram: Flow of studies through identification, screening, and inclusion. The original search (1 January 2015 through 30 September 2025) and an updated search (through 23 April 2026) identified 2164 records across PubMed, Embase, Web of Science, and CENTRAL. After removal of duplicates and screening of titles, abstracts, and full texts, seven publications reporting six trials met inclusion criteria [7,13,19,22,23,24,25]. The updated search yielded no additional eligible studies. SWIFT-Review was used for title and abstract screening.

The systematic search identified 2004 records across four electronic databases (PubMed: 188, Embase via Ovid: 842, Web of Science Core Collection: 231, CENTRAL: 743). After removing 429 duplicate records, 1575 unique records underwent title and abstract screening, of which 1520 were excluded. Full-text assessment was conducted for 55 articles, of which 48 were excluded for the following reasons: no extractable data (n = 16), wrong intervention (n = 8), conference abstract without sufficient data (n = 6), wrong comparator (n = 6), wrong study design (n = 5), wrong outcomes (n = 4), and wrong patient population (n = 3). The selection process is shown in Figure 1.

In the updated search conducted through 23 April 2026, 160 additional records were identified (CENTRAL: 72, Embase via Ovid: 25, PubMed: 15, Web of Science Core Collection: 48). After removal of 22 duplicate records, 138 unique records underwent title and abstract screening, of which 131 were excluded at that stage. Seven records were retrieved for full-text review, all of which were subsequently excluded for the following reasons: wrong comparator (n = 3), conference abstract without sufficient data (n = 1), wrong study design (n = 1), wrong intervention (n = 1), and no extractable data (n = 1). No additional studies met eligibility criteria.

Seven publications reporting six independent trials met inclusion criteria, including 268 unique participants (range: 12 to 131 per trial) [7,13,19,22,23,24,25]. Lugtenberg 2021 is a secondary analysis of the DIRECT trial (de Groot 2020); it used the same study population, contributes no additional participants, and was included because it reports validated QoL and patient-reported outcomes not reported in the primary publication [7,23]. Five trials (Bahrami 2024, DIRECT, Omar 2022, Riedinger 2020, Tang 2021; 255 participants) contributed continuous outcomes to the primary analysis, and five trials (Bahrami 2024, de Groot 2015, DIRECT, Omar 2022, Riedinger 2020; 256 participants) contributed adverse event outcomes [13,19,22,24,25]. Participant numbers refer to randomized participants; in DIRECT, 129 participants were analyzed for adverse events and 87 completed QoL questionnaires before the last cycle. Breast cancer was studied in four trials (Bahrami 2024, de Groot 2015, DIRECT, Omar 2022), gynecologic malignancies in one (Riedinger 2020), and lymphoma in one (Tang 2021) [13,19,22,24,25]. We evaluated five fasting protocols: FMDs (Bahrami 2024, DIRECT), STF (de Groot 2015), IF (Omar 2022), WOF (Riedinger 2020), and SCR (Tang 2021) [13,19,22,24,25]. All participants were receiving active systemic chemotherapy. Table 1 summarizes the characteristics of the included trials.

### 2.6. Outcome Organization and Dependency Structure

Patient outcomes were organized hierarchically to address within-study dependency while maximizing data utilization. Outcomes were classified by measurement type as continuous or dichotomous. Within each study, related outcomes were grouped into dependent effect sizes representing conceptually distinct biological or clinical constructs.

Dependent effect sizes were defined a priori based on biological and clinical rationale to ensure that outcomes within each group represented interchangeable measurements of the same underlying construct. For hematologic parameters, separate groups of effect sizes were created for red blood cell measures (RBC count and hemoglobin), white blood cell measures (WBC count and neutrophil count), and platelet measures. Metabolic markers were separated into glucose homeostasis parameters (glucose and insulin) and insulin-like growth factor-1 (IGF-1). Nutritional status indicators (prealbumin and BMI) were grouped together as related measures of nutritional state. QoL assessments, psychosocial measures, and toxicity outcomes were grouped according to their validated instrument domains.

To reduce within-study dependency, when dependency outcome groups contained multiple measurements of the same construct, one representative outcome was selected per dependent group of effect sizes. We retained the representative outcome using a fixed rule: the outcome listed first on the standardized extraction form. This rule did not follow a prespecified clinical hierarchy. Four of 15 continuous and six of 12 dichotomous dependency groups contained more than one candidate outcome. For the hematologic toxicity group in Riedinger 2020, we selected neutropenia instead of anemia, which is listed first in the trial’s table, so the hematologic toxicity outcome matched the other trials [13]. Because effect sizes from different dependency groups of the same trial remain correlated, this dependence was modeled in the synthesis (Section 2.7). To assess the influence of the selection rule, we repeated the analysis by selecting the last-listed outcome in each group and fitted a correlated, hierarchical-effects model that included all unique effect sizes with cluster-robust (CR2) variance estimation [26,27].

### 2.7. Effect Measures and Synthesis Methods

Effect sizes were calculated as the difference between the fasting intervention group and the non-fasting comparison group, such that positive values reflect outcomes favoring fasting. For continuous outcomes measured on different scales, standardized mean differences (SMDs) were calculated using Hedges’ g with small-sample bias correction [28]. Both continuous and dichotomous outcomes were identified across included studies. An initial attempt was made to harmonize all effect sizes through conversion of the originally extracted dichotomous risk ratios to standardized mean differences using the log-odds transformation [29]. However, conversion was deemed inappropriate due to several data limitations: a ceiling effect in Omar 2022 rendered the odds ratio for the nausea outcome mathematically undefined (100% control group event rate; the risk ratio was defined, RR = 0.71, 95% CI: 0.55–0.92, and was used in the adverse event model); of the 11 computable conversions, three required continuity correction for zero event cells; and converted standard errors (SEs) ranged from 0.215 to 0.912, reflecting imprecision attributable to small cell sizes (n = 10 per group in Riedinger 2020) [13,24]. Ten of the 11 converted 95% CIs crossed zero. Pooling these unstable converted values with continuous SMDs risked introducing bias and inflating variance estimates in the primary model. Accordingly, dichotomous adverse event outcomes were analyzed separately using log risk ratios in a three-level random-effects model (outcomes nested in trials) rather than being pooled with continuous SMDs. Converted SMD values with SEs are provided in Supplementary Table S1 for transparency but were not included in the primary analysis. Effect direction was coded such that positive values indicate benefit of fasting. We calculated Hedges’ g as the fasting group means minus the control group means, divided by the pooled standard deviation. For outcomes where lower values indicate a favorable state (glucose, insulin, IGF-1, C-reactive protein, fatigue, illness perception, and distress), we reversed the sign so positive values consistently favor fasting. For insulin, IGF-1, and glucose, we coded lower values as favorable because they reflect the mechanism proposed by the differential stress resistance hypothesis; they should not be interpreted as patient-important benefits. We coded higher blood cell counts as favorable because chemotherapy lowers them; for platelets, all arm means fell within the adult reference range of 150,000 to 450,000 per microliter [30], so higher values indicate less myelosuppression rather than thrombocytosis. The directions assigned to each outcome are reported in Supplementary Table S3. For dichotomous outcomes, risk ratios were calculated with continuity correction of 0.5 added to all cells when zero cells occurred.

Data were extracted as post-intervention means and SDs for each arm at the end of chemotherapy or, when follow-up differed between participants, at the latest time point with data for all participants. Values reported only at baseline, only within one group, or only as within-group change were not used. When SDs were not reported at follow-up, the baseline SDs reported in the same publication were used, as described in the Cochrane Handbook [31]; this applied to three outcomes from Lugtenberg 2021 [23]. Outcomes reported only as medians with interquartile ranges or ranges (metabolic and inflammatory markers in Bahrami 2024 and Omar 2022) were converted to means and SDs and analyzed only in sensitivity analyses, because the conversion assumes approximately normal distributions [22,24,32]. De Groot 2015 reported hematologic outcomes only as mean differences with 95% confidence intervals (CI) and figures, so these values were also analyzed only in sensitivity analyses [19]. Serum glucose in Riedinger 2020 was measured on cycle day 1 while participants in the fasting arm were still fasting; because it reflects adherence to the fast rather than a treatment effect, it was excluded [13]. For dichotomous outcomes, counts of patients with an event were used, and grade III or higher events were preferred when reported. When a publication reported conflicting values for the same outcome, the value less favorable to fasting was used in the primary analysis, and the alternative was examined in a sensitivity analysis.

Analyses were conducted in R version 4.5.2 (R Foundation for Statistical Computing, Vienna, Austria) [33] using the metafor package (version 4.8.0) [34] and the clubSandwich package (version 0.7.0) for cluster-robust inference [35]. The primary synthesis employed a three-level random-effects model in which effect sizes (level 2) were nested within independent trials (level 3), with sampling variance at level 1 [36]. Clustering was by independent trial rather than publication, so de Groot 2020 and Lugtenberg 2021, which report the same DIRECT participants, formed a single cluster [7,23]. We fitted the model using restricted maximum likelihood estimation. We used the Knapp–Hartung adjustment for small numbers of studies [37]. Because effect sizes from the same trial are correlated through shared participants, CR2 SE with Satterthwaite degrees of freedom were also calculated [26,27].

We quantified heterogeneity as total *I*^2^ and partitioned it into between-trial and within-trial components using the typical sampling variance [31,36], and we calculated a 95% prediction interval for the pooled estimate. Prespecified subgroup analyses examined whether effects differed by outcome domain, fasting type, cancer type, and systemic treatment type using moderator analysis [34]. Because the pooled SMD combined outcomes measured on different scales, estimates were also reported separately by outcome category (hematologic outcomes and QoL and patient-reported outcomes in the primary analysis; metabolic and inflammatory markers in a sensitivity analysis). We tested differences between categories and domains with omnibus moderator tests and applied a Bonferroni correction to the domain estimates. With five trials, we could not reliably estimate subgroup analyses by fasting type, cancer type, or treatment type, so we did not report them.

### 2.8. Sensitivity Analyses Methodology

Multiple sensitivity analyses assessed how analytic decisions affected the findings. Leave-one-cohort-out analysis systematically removed each independent trial and recalculated the pooled effect to identify influential observations. Additional analyses (1) excluded studies rated at high overall risk of bias; (2) fitted a two-level model with trial clusters; (3) added the metabolic and inflammatory values converted from medians; (4) added the de Groot 2015 values derived from CIs; (5) excluded outcomes with SDs taken from baseline; (6) excluded platelet outcomes; (7) selected the last-listed outcome in each dependency group; and (8) included all QLQ-C30 scales, or all unique effect sizes, in correlated and hierarchical-effects models with assumed within-trial correlations of 0.2, 0.4, 0.6, and 0.8 and CR2 SE [26,27]. For adverse events, analyses used the alternative DIRECT toxicity values, excluded the Riedinger 2020 hematologic toxicity counts, restricted outcomes to grade III or higher events, and included all unique outcomes.

### 2.9. Assessment of Publication Biases

Publication bias was assessed using a multilevel extension of Egger’s regression test, examining the relationship between effect sizes and their SEs by including the square root of sampling variance as a moderator in the three-level random-effects model. Because tests for funnel plot asymmetry are not recommended with fewer than 10 studies [38], the test was treated as exploratory and was repeated with CR2 SE and on effects aggregated to the trial level (assumed within-trial correlation of 0.6). Funnel plots displaying effect sizes against their SEs were generated and visually inspected for asymmetry, with interpretation conducted cautiously given fewer than 10 studies. Contour-enhanced funnel plots were used descriptively.

### 2.10. Risk of Bias Assessment Methodology

Two independent reviewers for each study (KMB, CGJ, MS, DT, LDR) independently assessed risk of bias using the revised Cochrane Risk of Bias (RoB) 2 tool for randomized trials, evaluating five domains: bias arising from the randomization process, bias due to deviations from intended interventions, bias due to missing outcome data, bias in measurement of the outcome, and bias in selection of the reported result [39]. Each domain was rated as low risk, some concerns, or high risk. For non-randomized studies, risk of bias was assessed using an approach aligned with ROBINS-I [40]. Judgments were displayed as a traffic-light plot and a summary plot following the robvis format [41].

## 3. Results

### 3.1. Data Structure and Outcome Organization

From the seven publications, we extracted 42 continuous and 22 dichotomous outcome measures (Supplementary Table S3). Eight further outcomes could not be analyzed: global QoL, insulin, and IGF-1 in de Groot 2020 (shown only in figures or Supplementary Tables), DNA damage in de Groot 2015 (SE of within-group comparisons), the FOSI-18 QoL score in Riedinger 2020 (no SD reported), serum glucose in Riedinger 2020 (measured during the fast), and prealbumin and BMI in Tang 2021 (no comparison group data at follow-up) [7,13,19,25].

For continuous outcomes, Bahrami 2024 and Omar 2022 each contributed hematologic outcomes (RBC count, hemoglobin, WBC count, neutrophils, and platelets) and metabolic and inflammatory markers (glucose, insulin, IGF-1, and C-reactive protein) reported as medians [22,24]. Lugtenberg 2021 contributed the EORTC QLQ-C30 scales, the Brief Illness Perception Questionnaire consequences item, and the distress thermometer, measured before the last chemotherapy cycle [23]. Riedinger 2020 contributed hemoglobin, absolute neutrophil count, and platelet count, and Tang 2021 contributed erythrocyte and leucocyte counts after the second fasting cycle [13,25]. De Groot 2015 reported erythrocyte and thrombocyte counts only as mean differences with 95% CIs [19].

For adverse events, Omar 2022 reported gastrointestinal toxicity (nausea, vomiting, diarrhea, and constipation) and mouth sores of any grade [24]; Bahrami 2024 reported grade III gastrointestinal, hematologic, and mucosal toxicity [22]; de Groot 2015 reported grade III/IV neutropenic fever, fatigue, and infection [19]; de Groot 2020 reported overall grade III/IV toxicity, neutropenia, and neutropenic fever [7]; and Riedinger 2020 reported any grade 3 or 4 toxicity, hematologic toxicity by grade, and neuropathy; grade 2 or higher neutropenia (four of 10 versus three of 10) was analyzed [13]. De Groot 2020 reported two different proportions of patients with grade III/IV toxicity: 75.4% versus 65.6% in the Results text and 47.7% versus 56.3% in Table 2 [7]. In Riedinger 2020, hematologic toxicity counts were shown under per-arm patient totals, but the accompanying text suggests that they were counted per chemotherapy cycle; they were entered as patient counts and examined in a sensitivity analysis [13].

To address within-study multiplicity, one representative outcome was selected from each dependency group when multiple measurements of the same construct existed. The primary analyses therefore included 15 continuous effect sizes from five trials and 12 adverse event outcomes from five trials.

### 3.2. Risk of Bias Assessment

**Figure 2:**
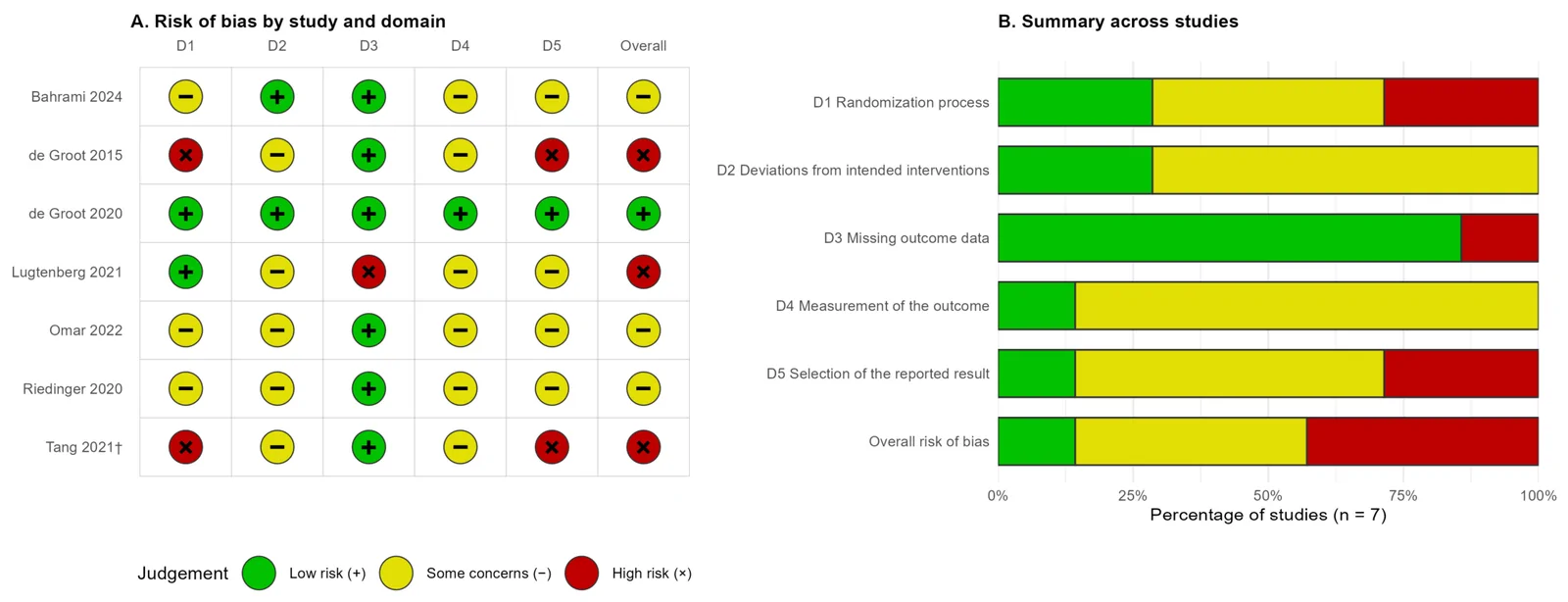
Risk of bias assessment. (**A**) Judgments by study and domain; (**B**) proportion of studies in each judgment category. Randomized trials were assessed with the revised Cochrane Risk of Bias tool (RoB 2) [39]; † Tang 2021 (non-randomized) was assessed with an approach aligned with ROB-INS-I [25,40]. Each judgment is shown with a color and a symbol (+, low risk; −, some concerns; ×, high risk) for legibility in grayscale. D1, randomization process; D2, deviations from intended interventions; D3, missing outcome data; D4, measurement of the outcome; and D5, selection of the reported result [7,13,19,22,23,24,25].

Risk of bias assessment revealed variable methodological quality (Figure 2). One publication (de Groot 2020) was rated at low overall risk of bias, three were rated as having some concerns (Bahrami 2024, Omar 2022, Riedinger 2020), and three were rated at high risk (de Groot 2015, Lugtenberg 2021, Tang 2021) [7,13,19,22,23,24,25]. De Groot 2015 was rated at high risk of bias because the methods of random sequence generation and allocation concealment were not reported and baseline characteristics differed between arms (stage III disease in 42.9% versus 16.7%), and because hematologic outcomes were reported only as day 7 values pooled across repeated measurements within patients, one of several possible analyses [19] (Figure 2). Tang 2021 was rated at high risk of bias because it was non-randomized, with a comparison group drawn from medical records [25]. Lugtenberg 2021 was rated at high risk of bias due to missing outcome data: questionnaire non-response before the last cycle was 41% in the fasting arm versus 23% in the control arm, and non-adherent patients were more likely to fail to complete questionnaires [23]. In addition, Omar 2022 replaced two participants who withdrew from the fasting arm, and Bahrami 2024 provided 2 g of omega-3 fatty acids daily to the fasting arm only [22,24].

### 3.3. Meta-Analysis of Continuous Outcomes

**Figure 3:**
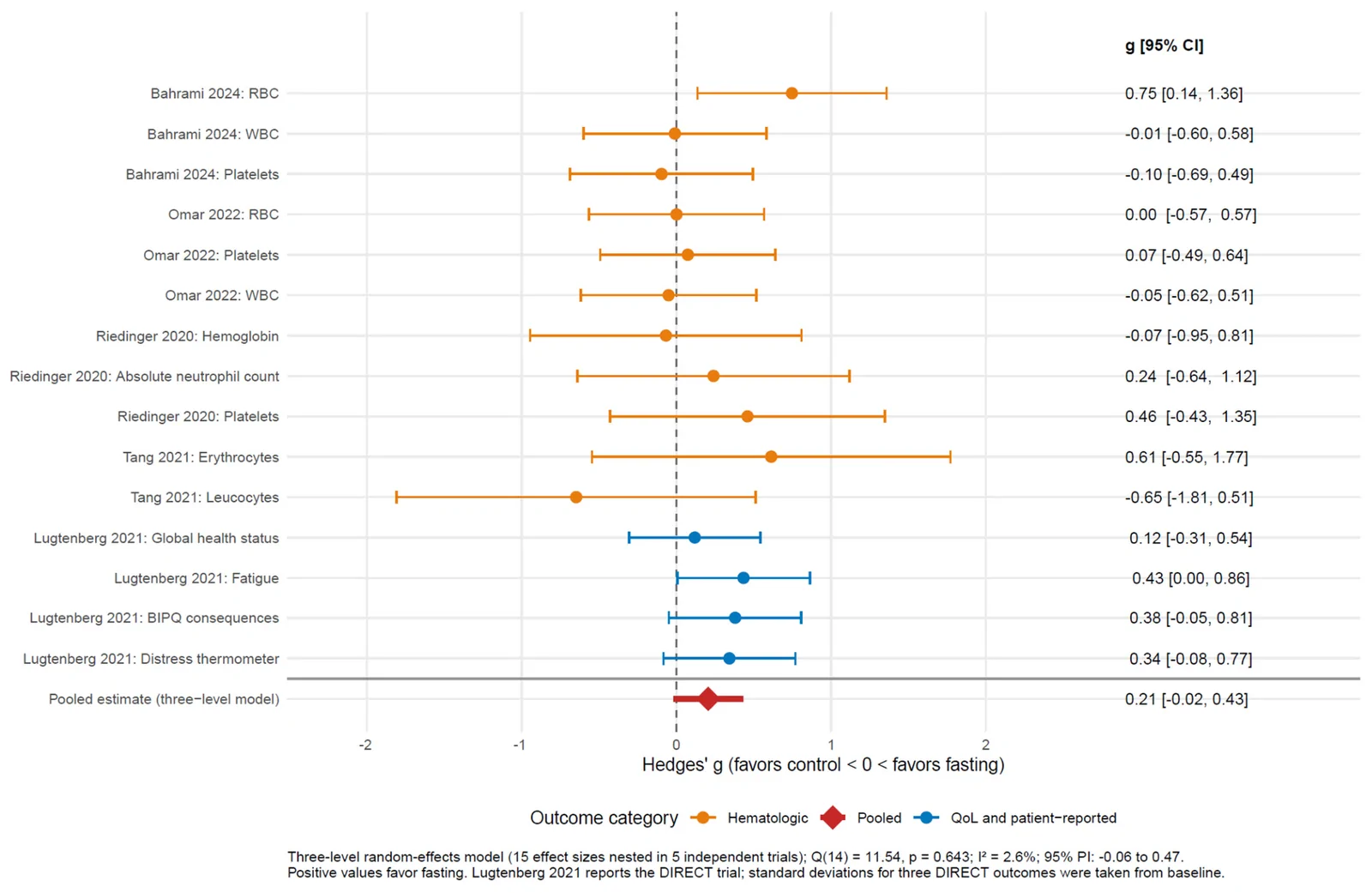
Forest plot of continuous health outcomes: Hedges’ g with 95% CIs for the 15 continuous outcomes from five independent trials in the primary analysis, synthesized using a three-level random-effects model with effect sizes nested within trials. Each point represents one effect size and is colored by outcome category (hematologic, or QoL and patient-reported outcomes). Horizontal lines indicate 95% CIs. The red diamond represents the pooled estimate (g = 0.21, 95% CI: −0.02–0.43, *p* = 0.064), which was not statistically significant. Lugtenberg 2021 reports the DIRECT trial [23]. Values to the right of the dashed reference line at zero favor fasting; values to the left favor the control condition [7,13,19,22,23,24,25].

The primary analysis included 15 effect sizes from five independent trials (Figure 3). The pooled SMD did not reach statistical significance (g = 0.21, 95% CI: −0.02–0.43, *p* = 0.064; CR2 *p* = 0.114). The point estimate was small and in the direction of fasting, yet both the 95% CI and the 95% prediction interval (−0.06–0.47) included no effect.

Heterogeneity was low (Q(14) = 11.54, *p* = 0.643; total *I*^2^ = 2.6%). Between-trial variance was small (τ^2^ = 0.002), and within-trial variance was estimated at zero.

Estimates did not differ significantly by outcome category (F(1, 3) = 1.86, *p* = 0.266; Table 2). Hematologic parameters did not differ between groups (g = 0.11, 95% CI: −0.23–0.45, *p* = 0.377; 11 effect sizes from four trials). QoL and patient-reported outcomes, all from the DIRECT trial, did not reach statistical significance (g = 0.32, 95% CI: −0.03 to 0.66, *p* = 0.062), although the point estimate favored fasting [7,23]. Metabolic and inflammatory markers were reported only as medians (Bahrami 2024 and Omar 2022); after conversion, the sensitivity analysis estimate was imprecise and not statistically significant (g = 0.62, 95% CI: −2.35–3.60, *p* = 0.229; six effect sizes from two trials) [22,24]. Because we coded lower glucose, insulin, IGF-1, and C-reactive protein values as favorable, this estimate reflects mechanistic changes rather than patient-important benefit.

### 3.4. Meta-Analysis of Dichotomous Outcomes

Twelve adverse event outcomes from five trials (256 participants) were analyzed (Supplementary Figure S1 and Table S5). Adverse events did not differ significantly between fasting and control groups (RR = 0.86, 95% CI: 0.55–1.36, *p* = 0.422), and heterogeneity was not statistically significant (Q(11) = 17.21, *p* = 0.102). Using the alternative DIRECT values from Table 2 gave RR = 0.78 (95% CI: 0.59–1.03, *p* = 0.067) [7,23]. Restricting the analysis to grade III or higher events gave RR = 0.88 (95% CI: 0.40–1.96, *p* = 0.647), and excluding the Riedinger 2020 hematologic toxicity counts gave RR = 0.84 (95% CI: 0.53–1.35, *p* = 0.374) [13]. Also, individual trials reported fewer gastrointestinal events with fasting, for example nausea in Omar 2022 (17 of 24 versus 24 of 24 participants; RR = 0.71, 95% CI: 0.55–0.92) and grade III vomiting in Bahrami 2024 (3 of 22 versus 12 of 22; RR = 0.25, 95% CI: 0.08–0.77), whereas grade III/IV toxicity in the DIRECT trial was numerically more frequent with fasting according to the Results text (75.4% versus 65.6%; RR = 1.15, 95% CI: 0.92–1.44) [7,22,23,24].

### 3.5. Subgroup Analyses

In the domain moderator model (Table 3), the estimate was g = 0.17 for physical outcomes (95% CI: −0.04–0.38, *p* = 0.098; 12 effect sizes from five trials), g = 0.12 for global QoL (95% CI: −0.35–0.59, *p* = 0.595), and g = 0.36 for psychosocial outcomes (95% CI: 0.02–0.70, *p* = 0.037; Bonferroni-corrected *p* = 0.112). Domains did not differ significantly (F(2, 12) = 0.65, *p* = 0.540). The global QoL and psychosocial estimates came from a single trial (DIRECT), and no functional outcome was included in the primary model because the baseline SDs of physical functioning, the representative functional outcome, reflected a ceiling effect; all functional scales were included in the all-scales sensitivity analysis.

The physical domain comprised 11 hematologic effect sizes and fatigue. Without fatigue, the estimate was that of the hematologic category (g = 0.11, 95% CI: −0.23–0.45, *p* = 0.377), so no outcome category produced a statistically significant physical domain effect. Metabolic and inflammatory markers were reported only as medians and were therefore analyzed only in sensitivity analyses.

### 3.6. Sensitivity Analyses

Leave-one-cohort-out estimates ranged from g = 0.11 to 0.26, and none was statistically significant (Supplementary Table S6). Excluding the trials rated at high risk of bias (Tang 2021 and the DIRECT outcomes reported by Lugtenberg 2021) gave g = 0.12 (95% CI: −0.35–0.59, *p* = 0.393) [23,25]. Adding the converted metabolic and inflammatory values gave g = 0.32 (95% CI: 0.07–0.56, *p* = 0.025). Adding the de Groot 2015 hematologic values gave g = 0.23 (95% CI: 0.03–0.43, *p* = 0.031) when the SD was derived from all measurements and g = 0.34 (95% CI: −0.19–0.88, *p* = 0.158) when it was derived from the number of patients [19]. Excluding outcomes with SDs taken from the baseline gave g = 0.16 (95% CI: −0.10–0.43, *p* = 0.166), excluding platelet outcomes gave g = 0.23 (95% CI: −0.01–0.47, *p* = 0.058), and selecting the last-listed outcome in each dependency group gave g = 0.17 (95% CI: −0.11–0.45, *p* = 0.163). Including all QLQ-C30 scales gave g = 0.16 to 0.18 (CR2 *p* = 0.078 to 0.087) across assumed within-trial correlations of 0.2 to 0.8, and including all 42 unique continuous effect sizes, including converted and derived values, gave g = 0.26 to 0.27 (CR2 *p* = 0.011 to 0.013). The direction of effect was consistent across analyses, yet statistical significance was reached only when values converted from medians or derived from CIs were added. Therefore, these sensitivity results should not be read as evidence of benefit.

### 3.7. Publication Bias Assessment

**Figure 4:**
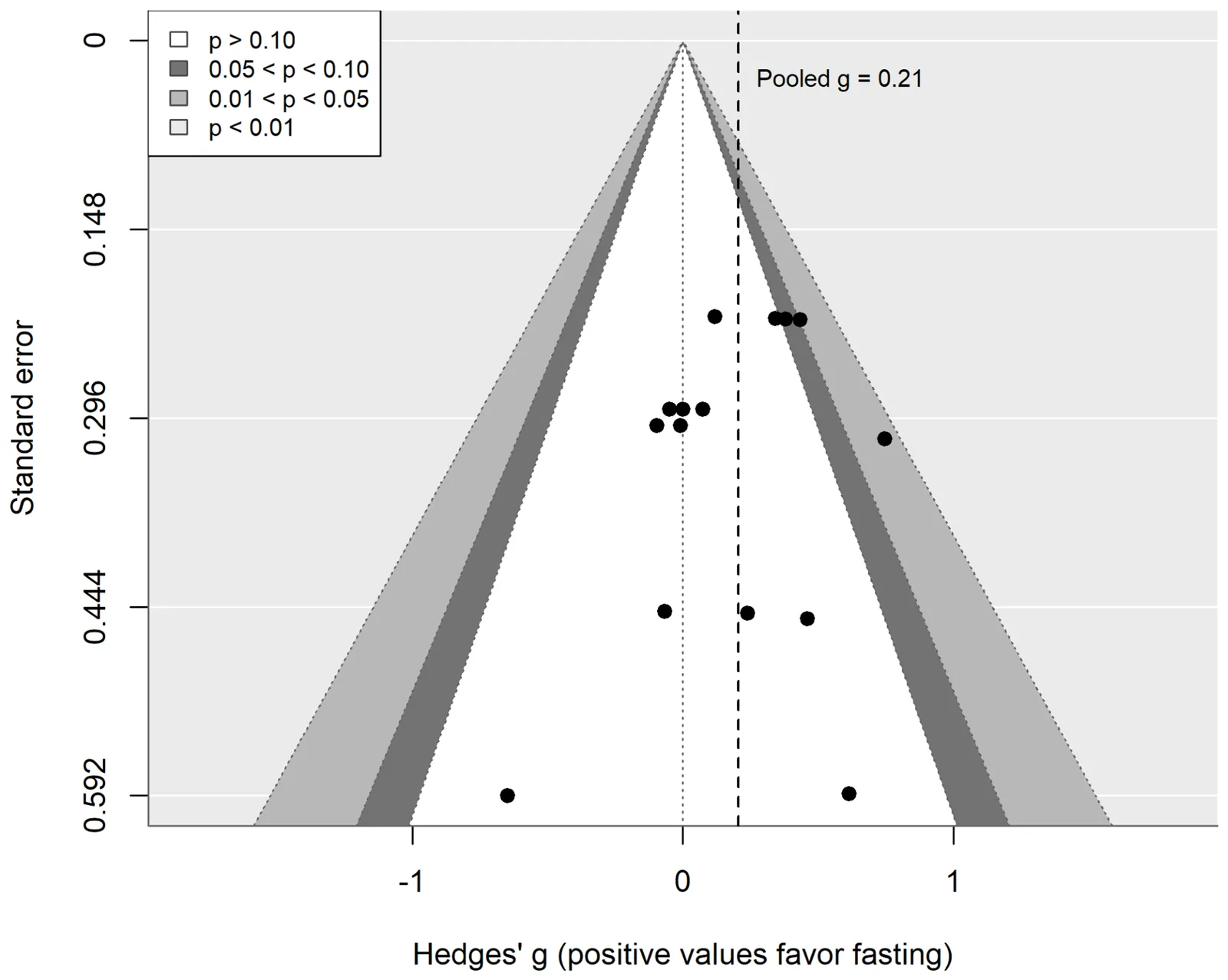
Funnel plot for assessment of publication bias: Contour-enhanced funnel plot of the 15 continuous effect sizes in the primary analysis (represented as the black dots) against their SEs. Shaded regions indicate significance contours centered at zero (*p* > 0.10, 0.05 < *p* < 0.10, 0.01 < *p* < 0.05, and *p* < 0.01). The dashed line indicates the pooled estimate from the three-level model (g = 0.21). Because fewer than 10 independent trials were available, the plot is presented for descriptive purposes; the multilevel Egger test showed no evidence of asymmetry (model-based *p* = 0.415; CR2 *p* = 0.329; trial-level *p* = 0.333) [7,13,19,22,23,24,25].

With five independent trials, formal tests for small-study effects have very limited power and reliability [38]. The multilevel Egger test showed no evidence of asymmetry in the model-based analysis (*p* = 0.415), with CR2 SE (*p* = 0.329), or when effects were aggregated to the trial level (*p* = 0.333; Supplementary Table S7). Figure 4 provides the funnel plot for descriptive purposes only.

## 4. Discussion

This meta-analysis examined fasting-based interventions during chemotherapy across hematologic, metabolic, and QoL outcomes. The pooled estimate was not statistically significant. Also, the overall pooled estimate combined hematologic parameters with patient-reported outcomes, which reflect different biological and clinical processes. Therefore, its clinical interpretation is limited; it is presented only as a summary of the direction of effect, and the category-specific estimates in Table 2 are the more informative results. Yet the point estimate was small and favored fasting, and heterogeneity was low. Hematologic parameters and adverse events did not differ between groups. QoL and patient-reported outcomes, available from a single trial, and metabolic markers, available only as medians from two trials, also did not differ significantly. With six small trials and wide CIs, these results neither confirm nor rule out a small benefit of fasting during chemotherapy. Therefore, they should be regarded as hypothesis-generating. Yet reporting inconclusive findings matters, because null and imprecise results are less likely to be published, which can bias the evidence toward benefit [42]. Hence, this review gives clinicians an accurate account of what the current trials can and cannot support.

In non-oncology populations, there is a body of literature that suggests IF can lower fasting glucose and insulin concentrations, improve insulin sensitivity and lipid metabolism, and reduce systemic inflammation [43]. Sutton et al. provided evidence that early time-restricted feeding can improve insulin sensitivity and blood pressure in men with prediabetes [44]. These findings are consistent in direction with the metabolic sensitivity analysis in the present review, but they come from populations not receiving chemotherapy and cannot be extrapolated to patients with cancer. Oncology-specific evidence remains somewhat limited and would benefit from larger, more comprehensive trials including RCTs. The existing oncology-specific literature varies across fasting protocols, cancer types, and chemotherapy regimens. Additionally, most of the trials included in this review that met the criteria were small and of moderate quality; only one met all low-risk criteria. These differences in protocols, populations, and study quality limit comparison across trials, although statistical heterogeneity was low in the present analysis.

De Groot et al. (2015) described short-term fasting as well tolerated, although two of seven patients stopped fasting after the third cycle [19]. In the DIRECT trial, grade III/IV toxicity did not differ significantly between groups, and no grade V toxicity occurred [7]. Riedinger et al. (2020) reported that a 48 h fast was well tolerated, although one participant withdrew because of feeling socially isolated while fasting [13], and Bahrami et al. (2024) reported that all patients completed eight cycles, with no grade IV or V toxicity in either group [22]. Yet adverse events were defined and graded differently across trials, the pooled RR was imprecise (RR = 0.86, 95% CI: 0.55–1.36), and the largest trial reported two conflicting proportions of grade III/IV toxicity, one numerically higher with fasting (75.4% versus 65.6%) and one lower (47.7% versus 56.3%). Therefore, these data are insufficient to establish the safety of fasting during chemotherapy, particularly for patients at nutritional risk, who were excluded from five of the six trials [7,13,19,22,23,24,25].

Fasting during chemotherapy raises concerns for patients at nutritional risk, because unintentional weight loss adversely affects clinical outcomes in patients with cancer, as noted in a prior systematic review of fasting during cancer treatment [45], and current guidance recommends regular assessment of nutritional intake, weight change, and BMI during treatment [46]. Five of six trials excluded patients at nutritional risk using BMI thresholds of 18.5 to 20.5 kg/m^2^, weight-loss limits, or albumin criteria; Bahrami 2024 did not report these criteria (Supplementary Table S4) [22]. Only three trials reported weight change by arm: in DIRECT, BMI decreased modestly with the FMD (median 0.33 kg/m^2^) and increased with a regular diet (median 0.64 kg/m^2^), whereas changes differed little between arms in Bahrami 2024 and Riedinger 2020 [13,22]; no trial described validated malnutrition screening during treatment. Adherence was limited in the largest trial, with 20.0% of participants following the FMD during all chemotherapy cycles. Fasting-related adverse effects were mild where reported (hunger and dizziness), one participant withdrew because of social isolation while fasting, and non-adherence in DIRECT was mainly attributed to dislike of components of the diet; adverse effects of fasting itself were not systematically assessed [7,23]. Therefore, these data do not establish that fasting is nutritionally safe during chemotherapy, and they do not apply to patients with cachexia, low BMI, or recent weight loss. Future trials should prespecify malnutrition screening, body composition, weight trajectories, and adherence as outcomes.

### 4.1. Strengths and Limitations

This meta-analysis represents the first quantitative synthesis examining fasting effects across multiple clinical domains in cancer patients receiving chemotherapy. Key strengths include the three-level random-effects modeling approach, which accounts for multiple effect sizes from the same trial, including shared participants across publications; verification of every extracted value against the source publications; sensitivity analyses of modeling, data handling, and outcome selection decisions; release of the extracted data and analysis code; comprehensive assessment of heterogeneity sources, and inclusion of both hematologic and patient-reported outcomes [31,36].

Several limitations warrant consideration. The evidence base is small and heterogeneous. The six independent trials enrolled between 12 and 131 participants, used five different fasting protocols, and predominantly enrolled patients with breast cancer (four of six trials). Also, only one publication (de Groot 2020) was rated at low overall risk of bias [7]. Analyzable QoL data were available from a single trial in which questionnaire completion differed between arms, adverse events were defined and graded differently across trials, and no trial reported long-term oncological outcomes such as progression-free or overall survival. With these limitations and the imprecision of the pooled estimates, the present findings should be regarded as hypothesis-generating. In addition, the overall pooled estimate combined laboratory and patient-reported outcomes, so it should not be interpreted as a single clinical effect.

Incomplete reporting in the primary studies required several analytic decisions. Metabolic and inflammatory markers were reported only as medians; SDs for three DIRECT outcomes were taken from baseline; de Groot 2015 reported hematologic outcomes only as mean differences with CIs; and the DIRECT trial reported two conflicting proportions of grade III/IV toxicity [19]. Statistical significance was reached only in sensitivity analyses that added converted or derived values, and the representative outcome in each dependency group was selected by a fixed rule rather than a prespecified clinical hierarchy. Also, Bahrami 2024 combined the FMD with omega-3 supplementation, and Omar 2022 replaced two participants who withdrew from the fasting arm [22,24].

None of the included trials reported outcomes by age group, race, or ethnicity, and participants were predominantly middle-aged (mean or median ages approximately 44 to 61 years). Therefore, the findings cannot be extended to older adults, who may be at greater nutritional risk during chemotherapy, and differences across demographic groups could not be examined. Participants were generally nourished, because five of six trials excluded patients at nutritional risk, and they may tolerate fasting differently from patients with cachexia, low BMI, or recent weight loss. Finally, the pooled SMD combines outcomes measured on different scales, limiting clinical interpretation; therefore, category-specific estimates are reported.

### 4.2. Clinical Implications

Because the SMD combined outcomes measured on different scales, it cannot be compared with a single minimal clinically important difference. The half standard deviation benchmark applies to health-related QoL measures [47], and the only analyzable QoL estimates, all from one trial, did not differ significantly between groups. We therefore describe magnitudes only with conventional statistical labels (small, moderate, large) and do not infer clinical importance from them.

The present evidence does not support clinical recommendations for fasting during chemotherapy. Hematologic parameters, QoL, and adverse events did not differ significantly between fasting and control groups, and the metabolic marker estimate depended on values converted from medians. Individual trials reported fewer gastrointestinal events with fasting. However, aggregate data cannot determine whether any symptomatic benefit is accompanied by better QoL, or whether QoL changes independently of symptoms. With the trials enrolling mainly well-nourished patients with breast cancer and excluding patients at nutritional risk, fasting during chemotherapy should be undertaken only within clinical trials or under the supervision of the treating oncology team, including nutritional assessment.

### 4.3. Future Directions

Several critical questions require investigation through adequately powered randomized trials. First, dose–response studies are necessary to establish optimal fasting duration and caloric thresholds. Second, cancer-specific trials are needed, particularly for malignancies with high metabolic demands or cachexia risk. Third, mechanistic studies incorporating serial biomarkers could identify predictive signatures for patient selection. Fourth, combination strategies with immunotherapy warrant exploration, given fasting’s immunomodulatory effects [18]. Finally, long-term follow-up is essential to assess impacts on treatment completion rates, disease recurrence, and survival. Future trials should report means and SDs for each arm at prespecified time points, grade adverse events with the CTCAE at the patient level, and prespecify patient-important outcomes, including chemotherapy dose intensity, dose delays and reductions, treatment completion, QoL, and validated nutritional outcomes, alongside long-term oncological outcomes. Until these trials are completed, we cannot determine whether fasting improves chemotherapy tolerance.

## 5. Conclusions

Across six small trials, fasting during chemotherapy did not produce statistically significant differences in hematologic parameters, QoL, or adverse events compared with a regular diet. Yet point estimates mostly favored fasting, and metabolic markers favored fasting only in a sensitivity analysis based on values converted from medians. With so few participants and wide CIs, a small benefit can neither be confirmed nor ruled out. Therefore, fasting during chemotherapy should be regarded as experimental and tested in adequately powered trials that report patient-important outcomes, standardized adverse events, and nutritional status.

## Figures and Tables

**Table 1:** Characteristics of included studies.

Study (Design; Country)	Participants, n (Fasting/Control)	Cancer Type; Chemotherapy	Fasting Protocol	Outcomes Used in This Review	Time Point Used
Bahrami 2024 (RCT; Iran) [22]	44 (22/22)	Breast, HER2-negative; neoadjuvant AC-T	FMD plus omega-3 ‡: 3 days before and on the day of chemotherapy; 8 cycles	Blood counts; metabolic markers ^a^; grade III toxicity	After cycle 8
de Groot 2015 (pilot RCT; Netherlands) [19]	13 (7/6)	Breast, HER2-negative; TAC	STF: 24 h before to 24 h after chemotherapy; 6 cycles	Erythrocytes, thrombocytes ^b^; grade III/IV toxicity	Day 7 after each cycle
DIRECT: de Groot 2020 (multicenter RCT; Netherlands) [7]	131 randomized; 129 analyzed (65/64)	Breast, HER2-negative; AC-T or FEC-T	FMD: 3 days before and on the day of chemotherapy	Grade III/IV toxicity; neutropenia	During treatment
DIRECT: Lugtenberg 2021 † (secondary analysis) [23]	87 with QoL data (38/49)	As for de Groot 2020	As for de Groot 2020	EORTC QLQ-C30; BIPQ consequences; distress thermometer	Before the last cycle
Omar 2022 (RCT; Egypt) [24]	48 (24/24)	Breast, HER2-negative; AC	IF: 18 h per day for 3 days per cycle; 4 cycles	Blood counts; metabolic markers ^a^; GI toxicity; mouth sores	After cycle 4
Riedinger 2020 (RCT; USA) [13]	20 (10/10)	Gynecologic; platinum-based	WOF: 24 h before to 24 h after chemotherapy	Hemoglobin, ANC, platelets; grade 3/4 toxicity; neutropenia; neuropathy	Cycle day 1 (laboratory values)
Tang 2021 (matched cohort; Taiwan) [25]	12 (6/6)	DLBCL; R-CHOP	SCR: 48 h water fast; 2 to 5 cycles	Erythrocytes, leucocytes	After the second fasting cycle

Note: All trials compared fasting with a standard (non-fasting) diet. Seven publications report six independent trials with 268 unique participants; de Groot 2020 and Lugtenberg 2021 report the same DIRECT trial [7,23]. ^a^ Reported as medians; converted to means and SDs and analyzed only in sensitivity analyses. ^b^ Reported as mean differences with 95% CIs; analyzed only in sensitivity analyses. † Same participants as de Groot 2020 [7,23]; 87 participants completed QoL questionnaires before the last cycle. ‡ The FMD arm also received 2 g of omega-3 fatty acids daily. Abbreviations: AC, doxorubicin (adriamycin) and cyclophosphamide; AC-T, AC followed by a taxane; ANC, absolute neutrophil count; BIPQ, Brief Illness Perception Questionnaire; DLBCL, diffuse large B-cell lymphoma; EORTC QLQ-C30, European Organisation for Research and Treatment of Cancer Quality of Life Questionnaire Core 30; FEC-T, fluorouracil, epirubicin, and cyclophosphamide followed by a taxane; FMD, fasting-mimicking diet; GI, gastrointestinal; IF, intermittent fasting; QoL, quality of life; R-CHOP, rituximab, cyclophosphamide, doxorubicin, vincristine, and prednisone; RCT, randomized controlled trial; SCR, short-term caloric restriction; STF, short-term fasting; TAC, docetaxel, doxorubicin (adriamycin), and cyclophosphamide; and WOF, water-only fasting.

**Table 2:** Pooled estimates by outcome category.

Outcome Category	k	Cohorts	g	95% CI	*p*
Hematologic (red cell, white cell, and platelet measures)	11	4	0.11	−0.23–0.45	0.377
QoL and patient-reported outcomes	4	1	0.32	−0.03–0.66	0.062
Metabolic and inflammatory (glucose, IGF-1, CRP) ^a^	6	2	0.62	−2.35–3.60	0.229

Abbreviations: CI, confidence interval; CRP, C-reactive protein; g, Hedges’ g (positive values favor fasting); IGF-1, insulin-like growth factor 1; k, number of effect sizes; Cohorts, number of independent trials; and QoL, quality of life. Hematologic and QoL estimates are from the primary three-level model with outcome category as a moderator (effect sizes nested in independent trials; Knapp–Hartung inference, *df* = 3). The test of differences between categories was F(1, 3) = 1.86, *p* = 0.266. QoL and patient-reported outcomes come from one trial (DIRECT) [7,23], and three of the four used SDs taken from baseline. Lower glucose, insulin, IGF-1, and CRP values were coded as favorable; these are mechanistic intermediates, not patient-important outcomes. ^a^ Sensitivity analysis only. These values were reported as medians with interquartile ranges or ranges and were converted to means and SDs [32]. The estimate is from a separate model of two trials. Note: No estimate in this table reached statistical significance (*p* < 0.05).

**Table 3:** Subgroup analysis by outcome domain.

Domain	Outcomes Included	k	Trials	g	95% CI	*p*	Bonferroni *p* †
Global	EORTC global health status	1	1	0.12	−0.94	0.595	1
Physical	Blood counts (11 effect sizes); fatigue	12	5	0.17	−0.42	0.098	0.293
Psychosocial	Illness perception (BIPQ consequences); distress	2	1	0.36	0.02–0.70	0.037 *	0.112
Functional	None estimable ^a^	0	0	–	–	–	–

Note: Estimates are from a three-level model with domain as a moderator (effect sizes nested in independent trials; Knapp–Hartung inference, *df* = 12); test of differences between domains: F(2, 12) = 0.65, *p* = 0.540. Domains: Global, overall QoL or general health status; physical, physical symptoms and bodily functioning, including laboratory measures of chemotherapy toxicity; psychosocial, emotional, social, and psychological well-being; and functional, ability to perform daily activities and roles. The global and psychosocial estimates come from a single trial (DIRECT) [7,23]. ^a^ Physical functioning, the representative functional outcome, was not analyzed because its baseline SDs reflected a ceiling effect; all functional scales were included in the all-scales sensitivity analysis. † Bonferroni correction for the three estimable domains (α = 0.05/3 = 0.0167). * *p* < 0.05. Abbreviations: BIPQ, Brief Illness Perception Questionnaire; CI, confidence interval; EORTC, European Organisation for Research and Treatment of Cancer; g, Hedges’ g (positive values favor fasting); k, number of effect sizes; and Trials, number of independent trials.

## Data Availability

This study is a meta-analysis of previously published research. All analyzed data were extracted from the published articles cited in the reference list and summarized in Table 1 and Supplementary Table S3. The complete extracted dataset (File S1) are provided as Supplementary Materials. No new primary data were generated.

## Supplementary Materials

The following supporting information can be downloaded at: https://www.mdpi.com/article/10.3390/nu18213681/s1, Table S1: Adverse event outcomes converted to standardized mean differences; Table S2: Full electronic search strategies; Table S3: Extracted data for every outcome, with source locations; Table S4: Nutritional eligibility criteria, body weight, adherence, and fasting-related effects in the included trials; Table S5: Adverse event outcomes: pooled estimates and sensitivity analyses; Table S6: Sensitivity analyses for the pooled continuous estimate; Table S7: Tests for small-study effects; Figure S1: Forest plot of adverse event outcomes in the primary analysis; File S1: extraction continuous and extraction dichotomous.

## References

[1] Bray F., Laversanne M., Sung H. (2024). Global cancer statistics 2022: GLOBOCAN estimates of incidence and mortality world-wide for 36 cancers in 185 countries. CA Cancer J. Clin..

[2] Ingrand I., Defossez G., Lafay-Chebassier C. (2020). Serious adverse effects occurring after chemotherapy: A general cancer registry-based incidence survey. Br. J. Clin. Pharmacol..

[3] Mariotto A.B., Yabroff K.R., Shao Y., Feuer E.J., Brown M.L. (2011). Projections of the Cost of Cancer Care in the United States: 2010-2020. JNCI J. Natl. Cancer Inst..

[4] Dorff T.B., Groshen S., Garcia A. (2016). Safety and feasibility of fasting in combination with platinum-based chemotherapy. BMC Cancer.

[5] Safdie F.M., Dorff T., Quinn D. (2009). Fasting and cancer treatment in humans: A case series report. Aging.

[6] Brandhorst S., Choi I.Y., Wei M. (2015). A Periodic Diet that Mimics Fasting Promotes Multi-System Regeneration, Enhanced Cognitive Performance, and Healthspan. Cell Metab..

[7] De Groot S., Lugtenberg R.T., Cohen D. (2020). Fasting mimicking diet as an adjunct to neoadjuvant chemotherapy for breast cancer in the multicentre randomized phase 2 DIRECT trial. Nat. Commun..

[8] Koppold-Liebscher D., Kessler C.S., Steckhan N. (2020). Short-term fasting accompanying chemotherapy as a supportive therapy in gynecological cancer: Protocol for a multicenter randomized controlled clinical trial. Trials.

[9] Kikomeko J., Schutte T., van Velzen M.J.M., Seefat R., van Laarhoven H.W.M. (2023). Short-term fasting and fasting mimicking diets combined with chemotherapy: A narrative review. Ther. Adv. Med. Oncol..

[10] Clifton K.K., Ma C.X., Fontana L., Peterson L.L. (2021). Intermittent fasting in the prevention and treatment of cancer. CA Cancer J. Clin..

[11] Stringer E.J., Cloke R.W.G., Van Der Meer L., Murphy R.A., Macpherson N.A., Lum J.J. (2025). The Clinical Impact of Time-restricted Eating on Cancer: A Systematic Review. Nutr. Rev..

[12] Nencioni A., Caffa I., Cortellino S., Longo V.D. (2018). Fasting and cancer: Molecular mechanisms and clinical application. Nat. Rev. Cancer.

[13] Riedinger C.J., Kimball K.J., Kilgore L.C., Bell C.W., Heidel R.E., Boone J.D. (2020). Water only fasting and its effect on chemotherapy administration in gynecologic malignancies. Gynecol. Oncol..

[14] Lee C., Raffaghello L., Brandhorst S. (2012). Fasting Cycles Retard Growth of Tumors and Sensitize a Range of Cancer Cell Types to Chemotherapy. Sci. Transl. Med..

[15] Raffaghello L., Lee C., Safdie F.M. (2008). Starvation-dependent differential stress resistance protects normal but not cancer cells against high-dose chemotherapy. Proc. Natl. Acad. Sci. USA.

[16] Raffaghello L., Safdie F., Bianchi G., Dorff T., Fontana L., Longo V.D. (2010). Fasting and differential chemotherapy protection in patients. Cell Cycle.

[17] Lee C., Safdie F.M., Raffaghello L. (2010). Reduced Levels of IGF-I Mediate Differential Protection of Normal and Cancer Cells in Response to Fasting and Improve Chemotherapeutic Index. Cancer Res..

[18] Cheng C.W., Adams G.B., Perin L. (2014). Prolonged Fasting Reduces IGF-1/PKA to Promote Hematopoietic-Stem-Cell-Based Regeneration and Reverse Immunosuppression. Cell Stem Cell.

[19] De Groot S., Vreeswijk M.P., Welters M.J. (2015). The effects of short-term fasting on tolerance to (neo) adjuvant chemotherapy in HER2-negative breast cancer patients: A randomized pilot study. BMC Cancer.

[20] Bauersfeld S.P., Kessler C.S., Wischnewsky M. (2018). The effects of short-term fasting on quality of life and tolerance to chemotherapy in patients with breast and ovarian cancer: A randomized cross-over pilot study. BMC Cancer.

[21] Howard B.E., Phillips J., Miller K., Tandon A., Mav D., Shah M.R., Holmgren S., Pelch K.E., Walker V., Rooney A.A. (2016). SWIFT-Review: A text-mining workbench for systematic review. Syst. Rev..

[22] Bahrami A., Haghighi S., Moghani M.M., Khodakarim N., Hejazi E. (2024). Fasting mimicking diet during neo-adjuvant chemotherapy in breast cancer patients: A randomized controlled trial study. Front. Nutr..

[23] Lugtenberg R.T., De Groot S. (2021). Quality of life and illness perceptions in patients with breast cancer using a fasting mimicking diet as an adjunct to neoadjuvant chemotherapy in the phase 2 DIRECT (BOOG 2013–14) trial. Breast Cancer Res. Treat..

[24] Omar E.M., Omran G.A., Mustafa M.F., El-Khodary N.M. (2022). Intermittent fasting during adjuvant chemotherapy may promote differential stress resistance in breast cancer patients. J. Egypt. Natl. Cancer Inst..

[25] Tang C.C., Huang T.C., Tien F.M., Lin J.M., Yeh Y.C., Lee C.-Y. (2021). Safety, Feasibility, and Effects of Short-Term Calorie Reduction during Induction Chemotherapy in Patients with Diffuse Large B-Cell Lymphoma: A Pilot Study. Nutrients.

[26] Hedges L.V., Tipton E., Johnson M.C. (2010). Robust variance estimation in meta-regression with dependent effect size estimates. Res. Synth. Methods.

[27] Tipton E. (2015). Small sample adjustments for robust variance estimation with meta-regression. Psychol. Methods.

[28] Hedges L.V. (1981). Distribution Theory for Glass’s Estimator of Effect size and Related Estimators. J. Educ. Stat..

[29] Hasselblad V., Hedges L.V. (1995). Meta-analysis of screening and diagnostic tests. Psychol. Bull..

[30] National Heart, Lung, and Blood Institute Thrombocytopenia. https://www.nhlbi.nih.gov/health/thrombocytopenia.

[31] Higgins J.P.T., Thompson S.G. (2002). Quantifying heterogeneity in a meta-analysis. Stat. Med..

[32] Wan X., Wang W., Liu J., Tong T. (2014). Estimating the sample mean and standard deviation from the sample size, median, range and/or interquartile range. BMC Med. Res. Methodol..

[33] R Core Team (2025). R: A Language and Environment for Statistical Computing.

[34] Viechtbauer W. (2010). Conducting Meta-Analyses in R with the metafor Package. J. Stat. Softw..

[35] Pustejovsky J.E. (2026). clubSandwich: Cluster-Robust (Sandwich) Variance Estimators with Small-Sample Corrections.

[36] Cheung M.W.L. (2014). Modeling dependent effect sizes with three-level meta-analyses: A structural equation modeling approach. Psychol. Methods.

[37] IntHout J., Ioannidis J.P., Borm G.F. (2014). The Hartung-Knapp-Sidik-Jonkman method for random effects meta-analysis is straightforward and considerably outperforms the standard DerSimonian-Laird method. BMC Med. Res. Methodol..

[38] Sterne J.A.C., Sutton A.J., Ioannidis J.P.A., Terrin N., Jones D.R., Lau J., Carpenter J., Rücker G., Harbord R.M., Schmid C.H. (2011). Recommendations for examining and interpreting funnel plot asymmetry in meta-analyses of randomised controlled trials. BMJ.

[39] Sterne J.A.C., Savović J., Page M.J. (2019). RoB 2: A revised tool for assessing risk of bias in randomised trials. BMJ.

[40] Sterne J.A., Hernán M.A., Reeves B.C. (2016). ROBINS-I: A tool for assessing risk of bias in non-randomised studies of interventions. BMJ.

[41] McGuinness L.A., Higgins J.P.T. (2021). Risk-of-bias VISualization (robvis): An R package and Shiny web app for visualizing risk-of-bias assessments. Res. Synth. Methods.

[42] Dwan K., Altman D.G., Arnaiz J.A., Bloom J., Chan A.W., Cronin E., Decullier E., Easterbrook P.J., Von Elm E., Gamble C. (2008). Systematic review of the empirical evidence of study publication bias and outcome reporting bias. PLoS ONE.

[43] Varady K.A., Cienfuegos S., Ezpeleta M., Gabel K. (2021). Cardiometabolic Benefits of Intermittent Fasting. Annu. Rev. Nutr..

[44] Sutton E.F., Beyl R., Early K.S., Cefalu W.T., Ravussin E., Peterson C.M. (2018). Early Time-Restricted Feeding Improves Insulin Sensitivity, Blood Pressure, and Oxidative Stress Even without Weight Loss in Men with Prediabetes. Cell Metab..

[45] Drexler U., Dörfler J., von Grundherr J., Erickson N., Hübner J. (2023). Fasting during cancer treatment: A systematic review. Qual. Life Res..

[46] Muscaritoli M., Arends J., Bachmann P., Baracos V., Barthelemy N., Bertz H., Bozzetti F., Hütterer E., Isenring E., Kaasa S. (2021). ESPEN practical guideline: Clinical nutrition in cancer. Clin. Nutr..

[47] Norman G.R., Sloan J.A., Wyrwich K.W. (2003). Interpretation of Changes in Health-related Quality of Life: The Remarkable Universality of Half a Standard Deviation. Med. Care.

