# Supplementary material for: Fasting During Chemotherapy and Hematologic, Metabolic, and Quality of Life Outcomes: A Systematic Review and Meta-Analysis
Source: Nutrients. 2026 Nov 6;18(21):3681. doi: 10.3390/nu18213681 (PMC13765235; doi:10.3390/nu18213681)
Supplement: Supplementary file 1 [file nutrients-18-03681-s001.zip › Supplementary materials.pdf]

Supplementary Table S1

Adverse event outcomes converted to standardized mean differences (not included in the primary analysis)

Study	Outcome	Grade	Fasting events/n	Control events/n	RR (95% CI)	SMD	SE	95% CI	Continuity correction
Omar 2022	Nausea	Any grade	17/24	24/24	0.71 (0.55 - 0.92)	Undefined †	–	–	No
Omar 2022	Mouth sores	Any grade	0/24	8/24	0.06 (0.00 - 0.97)	1.78	0.821	0.17 - 3.39	Yes
de Groot 2020	Any grade III/IV toxicity	Grade III/IV	49/65	42/64	1.15 (0.92 - 1.44)	-0.26	0.215	-0.68 - 0.16	No
de Groot 2020	Neutropenia	Grade III/IV	19/65	18/64	1.04 (0.60 - 1.79)	-0.03	0.215	-0.45 - 0.39	No
Bahrami 2024	Diarrhea	Grade III	3/22	5/22	0.60 (0.16 - 2.21)	0.34	0.443	-0.52 - 1.21	No
Bahrami 2024	Neutropenia	Grade III	5/22	10/22	0.50 (0.20 - 1.23)	0.57	0.367	-0.14 - 1.29	No
Bahrami 2024	Mouth sores	Grade III	7/22	10/22	0.70 (0.33 - 1.50)	0.32	0.346	-0.36 - 1.00	No
de Groot 2015	Neutropenic fever	Grade III/IV	2/7	2/6	0.86 (0.17 - 4.37)	0.12	0.664	-1.18 - 1.42	No
de Groot 2015	Fatigue	Grade III/IV	2/7	0/6	4.38 (0.25 - 76.54)	-0.98	0.912	-2.77 - 0.81	Yes
Riedinger 2020	Any grade 3 or 4 toxicity	Grade 3/4	0/10	2/10	0.20 (0.01 - 3.71)	1.00	0.891	-0.74 - 2.75	Yes
Riedinger 2020	Neutropenia	Grade 2 or higher	4/10	3/10	1.33 (0.40 - 4.49)	-0.24	0.521	-1.26 - 0.78	No
Riedinger 2020	Neuropathy	Any grade	3/10	2/10	1.50 (0.32 - 7.14)	-0.30	0.579	-1.43 - 0.84	No

Abbreviations: CI, confidence interval; OR, odds ratio; RR, risk ratio; SE, standard error; SMD, standardized mean difference.

Note. The 12 outcomes are those in the primary adverse event model. SMDs were obtained from the log odds ratio as $SMD = \ln(OR) \times \sqrt{3}/\pi$ (Hasselblad, 1995; reference [32] in the main text) and signed so that positive values favor fasting (fewer events with fasting). When a 2 x 2 table contained a zero cell, 0.5 was added to every cell. Risk ratios are those used in the adverse event model (Supplementary Table S5); RR < 1 indicates fewer events with fasting. †Nausea in Omar 2022 occurred in all 24 control participants, so the odds ratio is undefined without continuity correction and was not converted; the risk ratio is defined (RR = 0.71, 95% CI: 0.55 - 0.92) and was included in the adverse event model. Of the 11 converted outcomes, three required continuity correction, SEs ranged from 0.215 to 0.912, and 10 of 11 confidence intervals included zero. de Groot 2020 grade III/IV toxicity uses the Results text values (75.4% versus 65.6%).

Supplementary Table S2
Full electronic search strategies

Database	Search	Search string	Limits	Records
PubMed	Original	"cancer" AND ("fasting" OR "time-restricted eating" OR "time-restricted feeding" OR "Fasting-mimicking diet" OR "intermittent fasting" OR "alternative day fasting" OR "Short-term fasting" OR "Intermittent energy restriction") AND ("intervention" OR "treatment" OR "program" OR "clinical trial" OR "randomized controlled trial" OR "RCT" OR "cohort study") AND ("treatment outcomes" OR "quality of life" OR "fatigue" OR "progression-free survival" OR "CTCAE" OR "adverse events" OR "treatment completion rates") NOT ("after treatment" OR "post-treatment" OR "survivorship only" OR "preclinical study" OR "animal study")	2015 to 2025	188
Embase (Ovid)	Original	('cancer'/exp OR 'cancer') AND ('fasting'/exp OR 'fasting' OR 'time-restricted eating'/exp OR 'time-restricted eating' OR 'time-restricted feeding'/exp OR 'time-restricted feeding' OR 'fasting-mimicking diet'/exp OR 'fasting-mimicking diet' OR 'intermittent fasting'/exp OR 'intermittent fasting' OR 'alternative day fasting'/exp OR 'alternative day fasting' OR 'short-term fasting' OR 'intermittent energy restriction'/exp OR 'intermittent energy restriction') AND ('intervention'/exp OR 'intervention' OR 'treatment'/exp OR 'treatment' OR 'program'/exp OR 'program' OR 'clinical trial'/exp OR 'clinical trial' OR 'randomized controlled trial'/exp OR 'randomized controlled trial' OR 'rct' OR 'cohort study'/exp OR 'cohort study') AND ('treatment outcomes' OR 'quality of life'/exp OR 'quality of life' OR 'fatigue'/exp OR 'fatigue' OR 'progression-free survival'/exp OR 'progression-free survival' OR 'ctcae' OR 'adverse events'/exp OR 'adverse events' OR 'treatment completion rates') NOT ('after treatment' OR 'post-treatment' OR 'survivorship only' OR 'preclinical study'/exp OR 'preclinical study' OR 'animal study'/exp OR 'animal study')	2015 to 2025	842
Web of Science Core Collection	Original	"cancer" AND ("fasting" OR "time-restricted eating" OR "time-restricted feeding" OR "Fasting-mimicking diet" OR "intermittent fasting" OR "alternative day fasting" OR "Short-term fasting" OR "Intermittent energy restriction") AND ("intervention" OR "treatment" OR "program" OR "clinical trial" OR "randomized controlled trial" OR "RCT" OR "cohort study") AND ("treatment outcomes" OR "quality of life" OR "fatigue" OR "progression-free survival" OR "CTCAE" OR "adverse events" OR "treatment completion rates") NOT ("after treatment" OR "post-treatment" OR "survivorship only" OR "preclinical study" OR "animal study")	2015 to 2025	231
Cochrane CENTRAL	Original	"cancer" AND ("fasting" OR "time-restricted eating" OR "time-restricted feeding" OR "Fasting-mimicking diet" OR "intermittent fasting" OR "alternative day fasting" OR "Short-term fasting" OR "Intermittent energy restriction") AND ("intervention" OR "treatment" OR "program" OR "clinical trial" OR "randomized controlled trial" OR "RCT" OR "cohort study") AND ("treatment outcomes" OR "quality of life" OR "fatigue" OR "progression-free survival" OR "CTCAE" OR "adverse events" OR "treatment completion rates") NOT ("after treatment" OR "post-treatment" OR "survivorship only"	2015 to 2025	743

Database	Search	Search string	Limits	Records
		OR "preclinical study" OR "animal study") (All Text)		
PubMed	Update (April 23, 2026)	As above	Records after the original search	15
Embase (Ovid)	Update (April 23, 2026)	As above AND [30-09-2025]/sd NOT [23-04-2026]/sd	Date window shown	25
Web of Science Core Collection	Update (April 23, 2026)	As above	Publication years 2025 or 2026; document type Article	48
Cochrane CENTRAL	Update (April 23, 2026)	As above (All Text)	Last 9 months; trials only	72

Note. Strings reproduced from the search log. Free-text terms were used in PubMed, Web of Science, and CENTRAL; Emtree terms with explosion (/exp) were added in Embase. Original searches covered January 1, 2015, to September 30, 2025 (2,004 records); the update added 160 records.

Supplementary Table S3

Extracted data for every outcome, with source locations (machine-readable version: File S2)

Study	Outcome	Favorable	n (F/C)	Fasting: mean (SD) or events	Control: mean (SD) or events	g (SE) or RR	Source	Use
Bahrami 2024	RBC	Higher	22/22	4.06 (0.37)	3.79 (0.34)	0.75 (0.31)	Table 3	Primary
Bahrami 2024	Hemoglobin	Higher	22/22	12.15 (0.72)	12.21 (0.74)	-0.08 (0.30)	Table 3	Not selected
Bahrami 2024	WBC	Higher	22/22	4.55 (0.92)	4.56 (0.93)	-0.01 (0.30)	Table 3	Primary
Bahrami 2024	Neutrophils	Higher	22/22	3.98 (0.48)	3.58 (0.31)	0.97 (0.32)	Table 3	Not selected
Bahrami 2024	Platelets	Higher	22/22	316.77 (54.10)	322.04 (53.10)	-0.10 (0.30)	Table 3	Primary
Bahrami 2024	Glucose	Lower	22/22	97.50 (11.09)	106.00 (9.81)	0.80 (0.31)	Table 4 median (IQR)	Converted (sensitivity)
Bahrami 2024	Insulin	Lower	22/22	12.30 (5.48)	12.85 (8.92)	0.07 (0.30)	Table 4 median (IQR)	Converted (sensitivity)
Bahrami 2024	IGF-1	Lower	22/22	172.00 (21.79)	181.00 (18.03)	0.44 (0.31)	Table 4 median (IQR)	Converted (sensitivity)
Bahrami 2024	hs-CRP	Lower	22/22	3.49 (1.64)	5.15 (2.54)	0.76 (0.31)	Table 4 median (IQR)	Converted (sensitivity)
de Groot 2015	Erythrocytes (day 7)	Higher	7/6	MD = 0.37	SD = 0.54 (derived)	0.65 (0.57)	Results p. 4 (95% CI)	CI-derived (sensitivity)
de Groot 2015	Thrombocytes (day 7)	Higher	7/6	MD = 71.35	SD = 65.68 (derived)	1.01 (0.59)	Results p. 4 (95% CI)	CI-derived (sensitivity)
Lugtenberg	Global health status	Higher	38/49	61.50 (18.72)	59.20 (19.72)	0.12 (0.22)	Table 4; SD Table 3; SD	Primary

Study	Outcome	Favora ble	n (F/C)	Fasting: mean (SD) or events	Control: mean (SD) or events	g (SE) or RR	Source	Use
2021							from baseline	
Lugtenberg 2021	Fatigue	Lower	38/49	43.50 (24.65)	52.80 (18.26)	0.43 (0.22)	Table 4; SD Table 3; SD from baseline	Primary
Lugtenberg 2021	BIPQ consequences	Lower	38/49	5.91 (2.38)	6.79 (2.24)	0.38 (0.22)	Table 5	Primary
Lugtenberg 2021	Distress thermometer	Lower	38/49	5.53 (2.10)	6.27 (2.18)	0.34 (0.22)	Table 4; SD Table 3; SD from baseline	Primary
Lugtenberg 2021	Physical functioning	Higher	38/49	77.00 (6.44)	68.80 (11.13)	0.87 (0.23)	Table 4; SD Table 3; SD from baseline	All-scales sensitivity
Lugtenberg 2021	Role functioning	Higher	38/49	52.90 (22.08)	52.40 (17.78)	0.03 (0.22)	Table 4; SD Table 3; SD from baseline	All-scales sensitivity
Lugtenberg 2021	Emotional functioning	Higher	38/49	78.40 (20.59)	73.40 (20.17)	0.24 (0.22)	Table 4; SD Table 3; SD from baseline	All-scales sensitivity
Lugtenberg 2021	Cognitive functioning	Higher	38/49	75.50 (18.82)	70.50 (16.72)	0.28 (0.22)	Table 4; SD Table 3; SD from baseline	All-scales sensitivity
Lugtenberg 2021	Social functioning	Higher	38/49	68.60 (18.26)	62.80 (13.33)	0.37 (0.22)	Table 4; SD Table 3; SD from baseline	All-scales sensitivity
Lugtenberg 2021	Nausea	Lower	38/49	7.40 (12.92)	5.90 (11.91)	-0.12 (0.22)	Table 4; SD Table 3; SD from baseline	All-scales sensitivity
Lugtenberg 2021	Pain	Lower	38/49	25.50 (14.98)	22.90 (16.28)	-0.16 (0.22)	Table 4; SD Table 3; SD from baseline	All-scales sensitivity
Lugtenberg 2021	Dyspnea	Lower	38/49	20.60 (10.57)	26.40 (11.59)	0.52 (0.22)	Table 4; SD Table 3; SD from baseline	All-scales sensitivity
Lugtenberg 2021	Insomnia	Lower	38/49	39.20 (30.42)	37.50 (31.07)	-0.05 (0.22)	Table 4; SD Table 3; SD from baseline	All-scales sensitivity
Lugtenberg 2021	Appetite loss	Lower	38/49	21.60 (24.65)	29.90 (18.74)	0.38 (0.22)	Table 4; SD Table 3; SD from baseline	All-scales sensitivity
Lugtenberg 2021	Constipation	Lower	38/49	16.70 (13.48)	22.20 (21.41)	0.30 (0.22)	Table 4; SD Table 3; SD from baseline	All-scales sensitivity
Lugtenberg 2021	Diarrhea	Lower	38/49	13.70 (10.84)	10.40 (10.56)	-0.31 (0.22)	Table 4; SD Table 3; SD from baseline	All-scales sensitivity
Lugtenberg 2021	Financial difficulties	Lower	38/49	5.90 (12.51)	11.80 (9.44)	0.54 (0.22)	Table 4; SD Table 3; SD from baseline	All-scales sensitivity
Omar 2022	RBC	Higher	24/24	4.61 (0.53)	4.61 (0.72)	0.00 (0.29)	Table 4	Primary
Omar 2022	Hemoglobin	Higher	24/24	11.65 (1.27)	11.89 (1.30)	-0.18 (0.29)	Table 4	Not selected
Omar 2022	Platelets	Higher	24/24	383.60 (79.85)	376.04 (119.97)	0.07 (0.29)	Table 4	Primary

Study	Outcome	Favorable	n (F/C)	Fasting: mean (SD) or events	Control: mean (SD) or events	g (SE) or RR	Source	Use
Omar 2022	WBC	Higher	24/24	5.87 (1.87)	5.98 (2.33)	-0.05 (0.29)	Table 4	Primary
Omar 2022	Neutrophils	Higher	24/24	3.45 (1.46)	3.76 (1.65)	-0.20 (0.29)	Table 4	Not selected
Omar 2022	Glucose	Lower	24/24	99.25 (11.81)	149.65 (42.89)	1.58 (0.33)	Table 5 median (min-max)	Converted (sensitivity)
Omar 2022	Insulin	Lower	21/20	47.54 (43.85)	97.71 (56.20)	0.98 (0.33)	Table 5 median (min-max)	Converted (sensitivity)
Omar 2022	IGF-1	Lower	21/20	17.04 (13.19)	14.89 (10.64)	-0.18 (0.31)	Table 5 median (min-max)	Converted (sensitivity)
Omar 2022	CRP	Lower	22/19	5.08 (2.07)	5.83 (1.92)	0.37 (0.32)	Table 5 median (min-max)	Converted (sensitivity)
Riedinger 2020	Hemoglobin	Higher	10/10	10.80 (1.30)	10.90 (1.50)	-0.07 (0.45)	Table 2	Primary
Riedinger 2020	Absolute neutrophil count	Higher	10/10	4.70 (3.30)	4.00 (2.20)	0.24 (0.45)	Table 2	Primary
Riedinger 2020	Platelets	Higher	10/10	246.50 (96.50)	203.50 (82.50)	0.46 (0.45)	Table 2	Primary
Tang 2021	Erythrocytes	Higher	6/6	4.45 (0.29)	4.15 (0.57)	0.61 (0.59)	Table 2	Primary
Tang 2021	Leucocytes	Higher	6/6	5.84 (1.52)	7.48 (2.93)	-0.65 (0.59)	Table 2	Primary
Omar 2022	Nausea (Any grade)	Lower	24/24	17/24	24/24	RR = 0.71	Table 2	Primary
Omar 2022	Vomiting (Any grade)	Lower	24/24	2/24	19/24	RR = 0.11	Table 2	Not selected
Omar 2022	Diarrhea (Any grade)	Lower	24/24	0/24	12/24	RR = 0.04	Table 2	Not selected
Omar 2022	Constipation (Any grade)	Lower	24/24	8/24	0/24	RR = 17.00	Table 2	Not selected
Omar 2022	Mouth sores (Any grade)	Lower	24/24	0/24	8/24	RR = 0.06	Table 2	Primary
de Groot 2020	Any grade III/IV toxicity (Results text) (Grade III/IV)	Lower	65/64	49/65	42/64	RR = 1.15	Results text, p. 2	Primary
de Groot 2020	Any grade III/IV toxicity (Table 2) (Grade III/IV)	Lower	65/64	31/65	36/64	RR = 0.85	Table 2	Sensitivity (alternative value)
de Groot 2020	Neutropenia (Grade III/IV)	Lower	65/64	19/65	18/64	RR = 1.04	Table 2	Primary
de Groot 2020	Neutropenic fever (Grade III/IV)	Lower	65/64	5/65	5/64	RR = 0.98	Table 2	Not selected
Bahrami 2024	Diarrhea (Grade III)	Lower	22/22	3/22	5/22	RR = 0.60	Table 2	Primary
Bahrami 2024	Vomiting (Grade III)	Lower	22/22	3/22	12/22	RR = 0.25	Table 2	Not selected
Bahrami 2024	Nausea (Grade III)	Lower	22/22	4/22	9/22	RR = 0.44	Table 2	Not selected
Bahrami 2024	Constipation (Grade III)	Lower	22/22	1/22	3/22	RR = 0.33	Table 2	Not selected
Bahrami 2024	Neutropenia (Grade III)	Lower	22/22	5/22	10/22	RR = 0.50	Table 2	Primary
Bahrami 2024	Thrombocytosis (Grade III)	Lower	22/22	1/22	1/22	RR = 1.00	Table 2	Not selected
Bahrami 2024	Mouth sores (Grade III)	Lower	22/22	7/22	10/22	RR = 0.70	Table 2	Primary

Study	Outcome	Favorable	n (F/C)	Fasting: mean (SD) or events	Control: mean (SD) or events	g (SE) or RR	Source	Use
de Groot 2015	Neutropenic fever (Grade III/IV)	Lower	7/6	2/7	2/6	RR = 0.86	Table 2	Primary
de Groot 2015	Fatigue (Grade III/IV)	Lower	7/6	2/7	0/6	RR = 4.38	Table 2	Primary
de Groot 2015	Infection (Grade III/IV)	Lower	7/6	2/7	1/6	RR = 1.71	Table 2	Not selected
Riedinger 2020	Any grade 3 or 4 toxicity (Grade 3/4)	Lower	10/10	0/10	2/10	RR = 0.20	Table 2	Primary
Riedinger 2020	Neutropenia (Grade 2 or higher)	Lower	10/10	4/10	3/10	RR = 1.33	Table 2	Primary
Riedinger 2020	Anemia (Grade 2 or higher)	Lower	10/10	1/10	3/10	RR = 0.33	Table 2	Not selected
Riedinger 2020	Neuropathy (Any grade)	Lower	10/10	3/10	2/10	RR = 1.50	Table 2	Primary

Note. Favorable = direction of benefit (Higher: higher values favorable; Lower: lower values favorable, sign of g reversed). F/C = fasting/control. Time points: Bahrami 2024, after cycle 8; Lugtenberg 2021, before the last cycle (n = 38 vs 49 completers); Omar 2022, after cycle 4; Riedinger 2020, cycle day 1 averaged over cycles; Tang 2021, after the second fasting cycle; de Groot 2015, day 7 of cycles 1 to 5. Converted values: medians with IQR or range converted to means and SDs (Wan et al., 2014). CI-derived: mean difference (MD) and SD derived from the reported 95% CI of the difference, assuming 35 vs 30 measurements; with 7 vs 6 patients the SDs are smaller. Not analyzable and therefore not listed: de Groot 2020 global QoL, insulin, and IGF-1 (figures only); de Groot 2015 DNA damage (SEs of within-group comparisons); Riedinger 2020 FOSI-18 (no SD) and serum glucose (measured during the fast); Tang 2021 prealbumin and BMI (no comparison group follow-up). de Groot 2020 grade III/IV toxicity: the Results text (75.4% vs 65.6%) and Table 2 (31/65 vs 36/64) conflict; the less favorable value is used in the primary analysis. Riedinger 2020 hematologic toxicity counts were entered as patient counts although the text suggests cycle-level counts.

Supplementary Table S4

Nutritional eligibility criteria, body weight, adherence, and fasting-related effects in the included trials

Trial	Nutritional eligibility criteria	Weight or BMI during treatment	Adherence	Fasting-related effects and co-interventions
Bahrami 2024	None reported (excluded diabetes)	No between-group difference in weight or BMI after cycle 8	All 44 completed 8 cycles; compliance checked by food records and returned packets	FMD arm also received 2 g omega-3 daily; no grade IV or V toxicity
de Groot 2015	BMI ≥ 19 kg/m²	Baseline BMI only	2 of 7 stopped fasting after cycle 3 (pyrosis; recurrent febrile neutropenia)	"Well tolerated"; chemotherapy adjustments did not differ
DIRECT (de Groot 2020; Lugtenberg 2021)	BMI > 19 kg/m²; during the trial, weight loss > 10% was not permitted	BMI fell with FMD (median 0.33 kg/m² by end of therapy) and rose with regular diet (median 0.64 kg/m²)	81.5% completed cycle 1; 33.8% ≥ 4 cycles; 20.0% all cycles (21.5% in Lugtenberg 2021)	Non-adherence mainly due to dislike of diet components; discontinuation 27.7% vs 23.8%; no dexamethasone before AC/FEC in FMD arm
Omar 2022	Excluded BMI < 20.5 kg/m² or >	Baseline only (mean BMI about 32	2 participants withdrew from	Headache tabulated with all

Trial	Nutritional eligibility criteria	Weight or BMI during treatment	Adherence	Fasting-related effects and co-interventions
	10% weight loss in the prior year	kg/m²)	fasting after cycle 2 and were replaced	toxicities; not attributed to fasting
Riedinger 2020	BMI ≥ 18.5 kg/m²	Weight change -0.84 (SD = 2.33) vs -1.1 (SD = 3.55) kg (Table 2; the text reverses the arms)	95% of cycles completed with strict adherence; 3 patients ate a snack in one cycle	1 participant withdrew (felt socially isolated while fasting); dose reductions or delays in 5 vs 8 of 60 cycles
Tang 2021	Excluded BMI ≤ 18.5 kg/m² or low albumin	BMI rose in fasting arm (22.50 to 23.82 kg/m²); comparison group not reported	All completed 2 to 5 fasting cycles; all used rescue food (about 250 kcal per 24 h)	Mild hunger and dizziness in the first two cycles

Note. All entries verified against the full-text publications. BMI = body mass index; FMD = fasting-mimicking diet.

Supplementary Table S5

Adverse event outcomes: pooled estimates and sensitivity analyses

Analysis	k (trials)	RR	95% CI	p	Heterogeneity
Primary (one outcome per dependency group; DIRECT Results text)	12 (5)	0.86	0.55 - 1.36	0.422	Q(11) = 17.21, p = 0.102
DIRECT grade III/IV from Table 2	12 (5)	0.78	0.59 - 1.03	0.067	Q(11) = 9.97, p = 0.533
Excluding Riedinger 2020 hematologic counts	11 (5)	0.84	0.53 - 1.35	0.374	Q(10) = 16.82, p = 0.079
Grade III or higher events only	8 (4)	0.88	0.40 - 1.96	0.647	Q(7) = 7.28, p = 0.401
Grade III or higher only, DIRECT Table 2	8 (4)	0.80	0.48 - 1.33	0.262	Q(7) = 4.54, p = 0.716
All unique outcomes	22 (5)	0.80	0.47 - 1.35	0.299	Q(21) = 44.43, p = 0.002

Note. RR below 1 indicates fewer events with fasting. Three-level models (outcomes nested in trials), restricted maximum likelihood, Knapp-Hartung inference. A continuity correction of 0.5 was added to each cell when a zero cell occurred. Individual outcomes are listed in Supplementary Table S3 and Supplementary Figure S1.

Supplementary Table S6

Sensitivity analyses for the pooled continuous estimate

Analysis	k (trials)	g	95% CI	p	CR2 p
Primary analysis	15 (5)	0.21	-0.02 - 0.43	0.064	0.114
Two-level model, trial clusters	15 (5)	0.21	-0.02 - 0.43	0.064	0.114
Without Bahrami 2024	12 (4)	0.19	-0.15 - 0.53	0.169	0.235
Without DIRECT	11 (4)	0.11	-0.23 - 0.45	0.377	0.258
Without Omar 2022	12 (4)	0.26	-0.01 - 0.54	0.054	0.064
Without Riedinger 2020	12 (4)	0.20	-0.10 - 0.49	0.122	0.173

Analysis	k (trials)	g	95% CI	p	CR2 p
Without Tang 2021	13 (4)	0.21	-0.05 - 0.48	0.081	0.118
Excluding high risk of bias (Tang 2021; DIRECT outcomes from Lugtenberg 2021)	9 (3)	0.12	-0.35 - 0.59	0.393	0.288
Adding converted metabolic and inflammatory values	21 (5)	0.32	0.07 - 0.56	0.025	0.008
Metabolic and inflammatory values only (converted)	6 (2)	0.62	-2.35 - 3.60	0.229	–
Adding de Groot 2015 (SD from 35 vs 30 measurements)	17 (6)	0.23	0.03 - 0.43	0.031	0.071
Adding de Groot 2015 (SD from 7 vs 6 patients)	17 (6)	0.34	-0.19 - 0.88	0.158	0.152
Adding converted values and de Groot 2015	23 (6)	0.33	0.11 - 0.56	0.012	0.005
Excluding outcomes with SDs taken from baseline	12 (5)	0.16	-0.10 - 0.43	0.166	0.134
Excluding platelet outcomes	12 (5)	0.23	-0.01 - 0.47	0.058	0.117
Last-listed outcome per dependency group	15 (5)	0.17	-0.11 - 0.45	0.163	0.197
All QLQ-C30 scales, CHE, ϱ = 0.2	28 (5)	0.18	-0.07 - 0.43	–	0.087
All QLQ-C30 scales, CHE, ϱ = 0.4	28 (5)	0.17	-0.05 - 0.39	–	0.082
All QLQ-C30 scales, CHE, ϱ = 0.6	28 (5)	0.17	-0.04 - 0.37	–	0.080
All QLQ-C30 scales, CHE, ϱ = 0.8	28 (5)	0.16	-0.04 - 0.36	–	0.078
All unique effect sizes, CHE, ϱ = 0.2	42 (6)	0.27	0.13 - 0.42	–	0.012
All unique effect sizes, CHE, ϱ = 0.4	42 (6)	0.27	0.12 - 0.42	–	0.011
All unique effect sizes, CHE, ϱ = 0.6	42 (6)	0.26	0.11 - 0.41	–	0.012
All unique effect sizes, CHE, ϱ = 0.8	42 (6)	0.26	0.11 - 0.41	–	0.013

Note. Three-level models (effect sizes nested in independent trials), restricted maximum likelihood, Knapp-Hartung inference. CR2 = bias-reduced cluster-robust variance estimator with Satterthwaite degrees of freedom; CHE = correlated and hierarchical effects model with assumed within-trial correlation ϱ (95% CI and p from CR2). Converted values: medians with IQR or range converted to means and SDs (Wan et al., 2014).

Supplementary Table S7

Tests for small-study effects (exploratory)

Test	Units	p
Multilevel Egger regression, three-level model (t test)	15 effects, 5 trials	0.415
Same model with CR2 standard errors	15 effects, 5 trials	0.329
Egger regression on trial-level aggregated effects	5 trials	0.333

Note. Effects were aggregated assuming a within-trial correlation of 0.6. Tests for funnel plot asymmetry are not recommended with fewer than 10 studies (Sterne et al., 2011).

Supplementary Figure S1

Forest plot of adverse event outcomes in the primary analysis

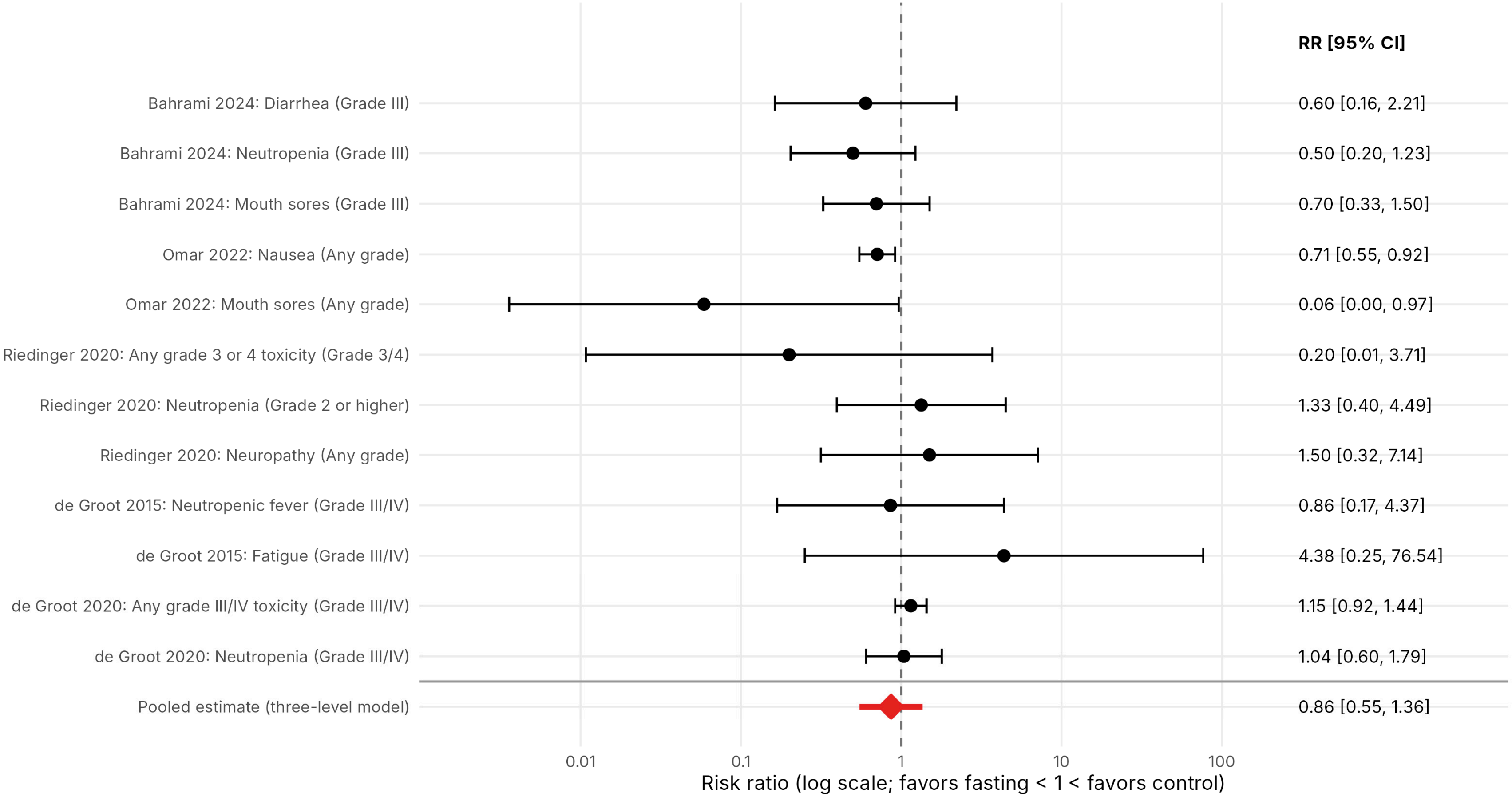

Risk ratios below 1 indicate fewer events with fasting. Three-level model (outcomes nested in trials): $Q(11) = 17.21$, $p = 0.102$.
de Groot 2020 grade III/IV toxicity: Results text (75.4% vs 65.6%), the less favorable of two conflicting reports
(Table 2: 31/65 vs 36/64). Riedinger 2020 neutropenia entered as patient counts; the text suggests cycle-level counts.
